# Synthesis of Cyclodextrin-Based Multifunctional Biocompatible Hydrogels and Their Use in the Prevention of Intrauterine Adhesions (Asherman’s Syndrome) after Surgical Injury

**DOI:** 10.1021/acsomega.4c03655

**Published:** 2024-07-11

**Authors:** Busra Aksoy Erden, Meltem Kurus, Ilgin Turkcuoglu, Rauf Melekoglu, Sevgi Balcioglu, Birgul Yigitcan, Burhan Ates, Suleyman Koytepe

**Affiliations:** † Central Research Laboratory Application and Research Center, Bartın University, Bartin 74110, Turkey; ‡ Faculty of Medicine, Department of Histology and Embryology, İzmir Katip Çelebi University, Izmir 35620, Turkey; § Faculty of Medicine, Department of Obstetrics and Gynecology, SANKO University, Gaziantep 27090, Turkey; ∥ Faculty of Medicine, Department of Obstetrics and Gynecology, İnönü University, Malatya 44280, Turkey; ⊥ Department of Medicinal Laboratory, Sakarya University of Applied Sciences, Sakarya 54050, Turkey; # Faculty of Science and Literature, Department of Chemistry, İnönü University, Malatya 44280, Turkey; ¶ Faculty of Medicine, Department of Histology and Embryology, İnönü University, Malatya 44280, Turkey

## Abstract

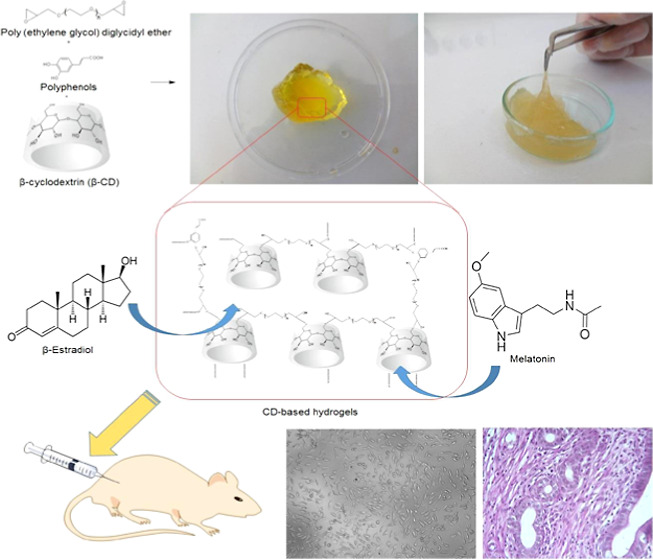

Asherman’s syndrome, which can occur during the regeneration of damaged uterine tissue after surgical interventions, is a significant health problem in women. This study aimed to acquire and characterize cyclodextrin-based hydrogels, which can be used to prevent Asherman’s syndrome, and investigate their effectiveness with biomedical applications. A series of hydrogels were synthesized from the cross-linking of β-cyclodextrin and different polyphenols with epoxy-functional PEG. Their chemical, physical, and biological properties were subsequently determined. The results demonstrated that the cyclodextrin-based hydrogels had a porous structure, high swelling ratio, good injectability, drug release ability, and antioxidant activity. Cell culture results illustrated that the hydrogels had no significant cytotoxicity toward L929 fibroblast cells. Considering all properties, the β-CD-PEG-600-Ec hydrogel showed the most satisfactory properties rather than other ones. The potential of this hydrogel in preventing Asherman’s syndrome was evaluated in a rat model. The results revealed that the β-estradiol- and melatonin-loaded cyclodextrin-based multifunctional hydrogel group both structurally and mechanically showed an antiadhesion effect in the uterus and a therapeutic effect on the damage with the β-estradiol and melatonin that it contains compared to the Asherman (ASH) group. This double drug-loaded hydrogel can be a promising candidate for preventing Asherman’s syndrome due to its versatile properties.

## 1 Introduction

**Figure 1:**
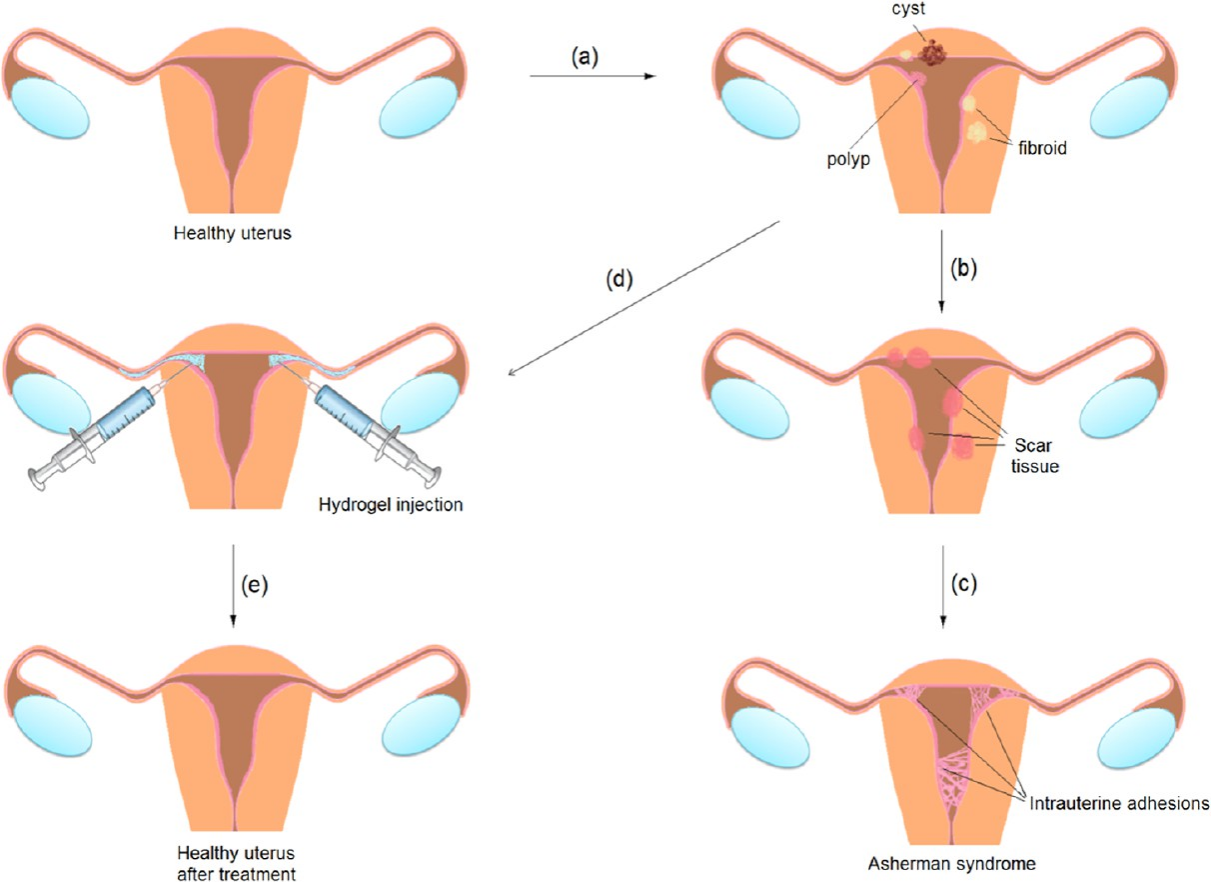
Formation of Asherman’s syndrome and the hydrogel treatment method.

Asherman’s syndrome, also *known as* intrauterine adhesions, is one of the most common diseases in women.^1−3^ The general consequences of Asherman’s syndrome are infertility, menstrual irregularities, pelvic pain, and recurrent pregnancy loss. Asherman’s syndrome may occur in many conditions that require intrauterine surgery.^1^ During birth, cesarean, curettage, removal of uterine cancer tissue, uterine fibroids, and polyp, intrauterine bleeding, infections, removal of intrauterine cysts (fibro cysts, fibroadenomas, chocolate cysts), and in postmenopausal periods, damage in the uterine walls can occur spontaneously^4^ (Figure 1a). The probability of these damages increases considerably with age and weight gain in women. After the operations performed in the uterus and ovary canal, cut areas are formed. The resulting damage can lead to the destruction of some layers of the endometrium and damage to the uterine muscle layer.^5^ As these wounds heal, scar tissue forms (Figure 1b), and sometimes, because this area is too narrow, scar tissue (scar crust tissue) adjoins and intrauterine adhesions may occur (Figure 1c).^1^ Today, there is no treatment method other than surgical intervention^4^ and hysteroscopy^6^ to treat and prevent this negative case. The development of new methods, which will provide an alternative to prevent any risks after surgical interventions in the uterus, may help the treatment in the relevant process. For example, designing an injectable hydrogel and applying it to the relevant area (Figure 1d) to prevent Asherman’s syndrome after surgery to be performed in the uterus may prevent the uterine and ovarian ducts from sticking together. In addition, the process can be completed when the biodegradable hydrogel applied after tissue healing decomposes and moves away from the relevant area (Figure 1e). In this way, it is aimed to prevent or treat Asherman’s syndrome. Hydrogels are cross-linked, hydrophilic, three-dimensional network polymers that can hold significant amounts of water.^7^ They are widely used in the pharmaceutical and biomedical fields due to their excellent and tunable chemical and physical properties.^8−14^ The hydrogel structure to be obtained can be considered a safe method that can be used as a precaution to prevent Asherman’s syndrome in many cases such as birth, cesarean section, curettage, removal of uterine cancer tissue, uterine fibroids, polyp, and intrauterine cysts, intrauterine bleeding, and intrauterine examinations.^15^

Treatment approaches with the hydrogel for uterine diseases and injuries are limited in the literature. In these studies, hydrogels containing poly(vinyl alcohol)/carboxymethylcellulose,^16^ polyzene,^17^ (poly(ε-caprolactone)-poly(ethylene glycol)-poly(ε-caprolactone)),^18^ poly(amidoamine) dendrimer-PEG,^19^ gelatin/PEG,^20^ chitosan,^21^ methylcellulose,^22^ liposome,^23^ hyaluronic acid,^24^ 17β-estradiol heparin-poloxamer,^25^ and chitosan-heparin hydrogel^26^ have been employed. Especially, to prevent intrauterine adhesion, different hydrogel structures have been tried in many academic studies. However, these studies are still continuing to find the most suitable hydrogel structure. Especially high swelling of the hydrogel structure to be applied may create a foreign body effect inside the uterus. Moreover, if the hydrogel structure is toxic or allergic, it may cause some serious problems. For this reason, studies continue to find the most suitable hydrogel structure. Recently, gel structures such as heparin-poloxamer, aloe/poloxamer, collagen, and hyaluronic acid have been used to prevent intrauterine adhesion.^25−32^ However, no literature has been found on cyclodextrin-based hydrogels to prevent uterine damage and associated Asherman’s syndrome.

**Figure 2:**
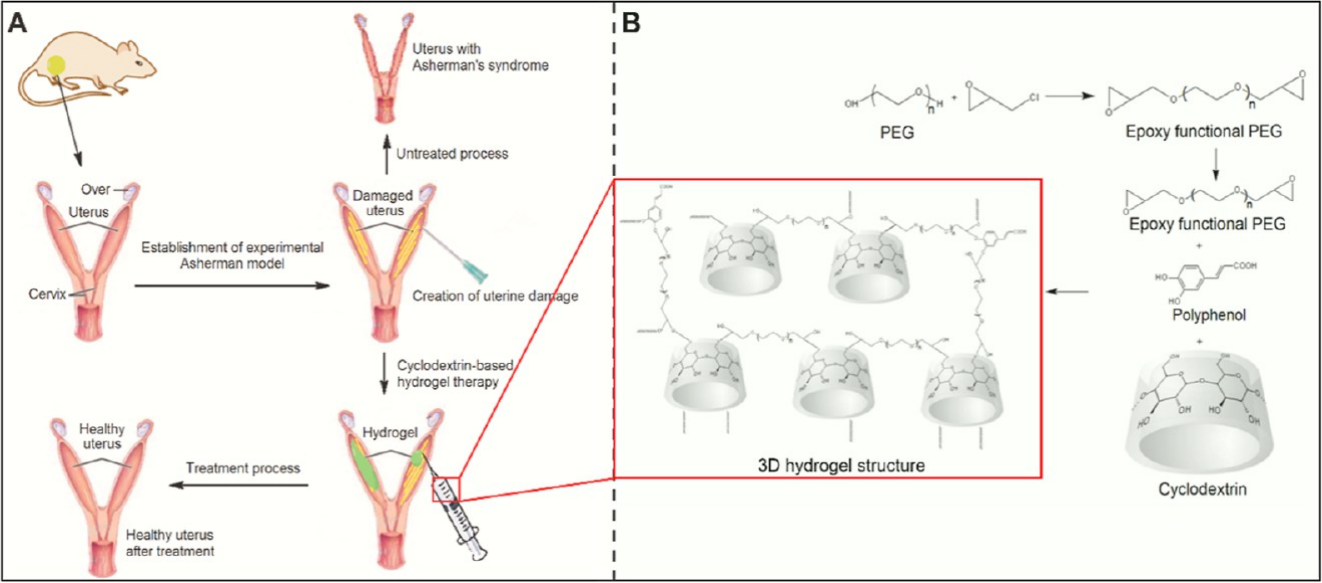
(A) Design of an experimental Asherman rat model and schematic representation of hydrogel treatment. (B) Schematic representation of the hydrogel structures.

Here, biocompatible, injectable, with good antiadhesion barrier ability, drug-releasing, antioxidant cyclodextrin-based hydrogels to be used to prevent possible Asherman’s syndrome after intrauterine surgery have been proposed. These hydrogels contain β-estradiol and melatonin-loaded cyclodextrin-based hydrogels that can eliminate oxidative stress in the uterine, improve uterine damage, and prevent intrauterine adhesions. First, hydrogels were prepared using epoxy-functional PEG, β-cyclodextrin, and different polyphenols. The hydrogels’ structural, morphological, and thermal characterizations were confirmed by FTIR, SEM, and TGA-DSC techniques, respectively. Their swelling ratio, injectability, *in vitro* biocompatibility, drug release properties, and antioxidant activity were evaluated, and their most promising application formulation was determined for *in vivo* studies. Then, the drug-loaded hydrogel was injected into the uterine horns of rats created with Asherman’s syndrome (Figure 2A), and its potential in preventing Asherman’s syndrome was elucidated. To the best of our knowledge, such a matrix containing the cyclodextrin-based hydrogel has not been utilized to prevent intrauterine adhesions yet. The aim of the study is to synthesize, characterize, and determine the *in vivo* efficiency of cyclodextrin-based hydrogels at different pore size and cross-linking ratios, which can be used in postintrauterine surgical interventions and reconstitution of damaged uterine tissue to prevent possible intrauterine adhesions. Therefore, cyclodextrin-based injectable hydrogels with mechanical barrier properties have been synthesized to prevent the formation of Asherman’s syndrome with this study. These hydrogel structures were prepared from natural and nontoxic monomeric units in order to be injectable into the uterine horns. The structures of these hydrogels consist of PEG, polyphenols, and cyclodextrin groups as cross-linkers (Figure 2B). For the synthesis of injectable, drug-carrying, and mechanical barrier hydrogels, first the synthesis of epoxy functional (diglycidylether) structures with the PEG structure epichlorohydrin was carried out. In the second step, hydrogel synthesis was carried out by interacting diglycidylether structures with β-cyclodextrin and polyphenol structures. Two basic pores were created in the structures of the synthesized hydrogels to absorb melatonin and β-estradiol structures. In this way, hydrogels will both prevent the formation of intrauterine adhesions by acting as a mechanical barrier and will contribute to wound healing by releasing the drugs they carry.

## 2 Materials and Methods

### 2.1 Chemicals

PEG-600, β-cyclodextrin (β-CD), and polyphenols (PPhs) [caffeic acid (CA), gallic acid (GA), epicatechin (Ec), quercetin (Quer), and curcumin (Cur)] used in the synthesis were purchased from Merck, TCI and, Sigma-Aldrich, respectively. Melatonin and β-estradiol used for drug release were acquired from TCI. Sodium hydroxide, dimethyl sulfoxide (DMSO), 3-[4,5-dimethylthiazol-2-yl]-2,5-diphenyltetrazolium bromide (MTT), disodium hydrogen phosphate, and sodium dihydrogen phosphate were supplied from Merck, and methanol and ethanol were obtained from Sigma-Aldrich.

### 2.2 Synthesis of Hydrogels

PEG used in synthesizing hydrogels had an average molecular weight of 600 g mol^–1^. A two-step synthesis method was used to synthesize hydrogel structures. In the first step, epoxy-functional PEG was obtained from the reaction of the PEG-600, which will form a large part of the hydrogel structure with epichlorohydrin. Then, in the second step, ring-opening polymerization was performed with epoxy-functional PEG, β-CD, and different PPh types (Figure 2B).

#### 2.2.1 Synthesis of the Poly(ethylene glycol) 600 Diglycidyl Ether Structure

Poly(ethylene glycol) diglycidyl ether (PEG-600-DGE) was synthesized from the reaction of epichlorohydrin and PEG-600. According to the method, 0.1 mol of PEG-600 was mixed with 0.6 mol of NaOH and 2.4 g of water. To this solution, 0.6 mol of epichlorohydrin was added dropwise and slowly at a temperature, not above 45 °C. Then, the mixture was stirred for 2 h by adjusting the temperature to 40 °C. The organic solution was filtered, washed with dichloromethane, and concentrated in vacuum.^34,35^^1^H NMR (CDCl_3_, 300 MHz): δ = 3.67–3.71 (m, 2H), 3.54–3.57 (m, 52.92H), 3.28–3.34 (m, 2H), 3.03–3.08 (m, 2H), 2.65–2.70 (t, 2H), 2.49–2.52 (m, 2H); ^13^C NMR (CDCl_3_, 75 MHz): δ = 71.86, 70.48, 50.70, 44.10; EA: C 54.11% (calculated 54.29%) and H 8.99% (calculated 8.48%).

#### 2.2.2 Determination of Monomer Ratios and Temperature Optimization in Hydrogel Synthesis

In order to optimize the monomer ratios to be used before the synthesis studies, PEG-600-DGE, β-CD, and PPhs were tested at different ratios. First, the molar ratios of PEG-600-DGE, β-CD, and PPhs were studied as 96:2:2, 90:3:5, 92:4:4, and 90/4/6 (Figure S1). The determined optimum monomer ratio of PEG-600-DGE/β-CD/PPhs was 90/4/6. The effect of temperature on hydrogel synthesis was also investigated. The synthesis temperatures were tested as 20, 30, 40, 50, 60, 70, and 80 °C (Figure S2). As a result of this study, the desired hydrogel structure was achieved at 60 °C as the optimum temperature.

#### 2.2.3 Synthesis of β-CD-Based Hydrogels

β-CD-based hydrogels were obtained from the ring-opening polymerization of PEG-600-DGE with β-CD and different PPhs. CA, GA, Ec, Quer, and Cur were used as PPhs in the synthesis. The names and contents of the synthesized hydrogels are presented in Table 1. The synthesis of the obtained hydrogels is given schematically in Figure 2B. According to the synthesis method, 9.0 mmol PEG-600-DGE was mixed with 0.6 mmol CA dissolved in DMSO in the presence of concentrated NaOH solution for 1 h. Then, 0.4 mmol of β-CD was added, and the reaction was refluxed at 60 °C for 24 h. At the end of the reaction, different colored hydrogels were obtained, they were washed with distilled water and ethanol, and left to dry. The same procedure was repeated with other PPhs to synthesize other hydrogels.^36^ All hydrogels obtained were dialyzed with ethanol and pure water for 2 days and 5 days, respectively, to remove monomer residues before analysis. A dialysis tubing cellulose membrane (Sigma-Aldrich, avg. flat width 33 mm, molecular weight cutoff = 14,000) was used for this procedure. Alcohol and water for washing were replaced daily with fresh ones. Since dehydration of hydrogels is difficult under normal conditions, they were first frozen at −20° for 2 days and then lyophilized with a freeze-dryer (Armfield) for 24 h.

**Table 1:** Names and Molar Ratios of the Hydrogels Synthesized in the Study

	PEG-600-DGE	β-CD	PPh	PPh type
β-CD-PEG-600-CA	90	4	6	CA
β-CD-PEG-600-GA	90	4	6	GA
β-CD-PEG-600-Ec	90	4	6	Ec
β-CD-PEG-600-Quer	90	4	6	Quer
β-CD-PEG-600-Cur	90	4	6	Cur

### 2.3 Characterization of Poly(ethylene glycol) Diglycidyl Ether and Hydrogels

Fourier transform infrared spectroscopy (FTIR; PerkinElmer Spectrum Two), Elemental Analysis (EA; LECO CHNS-932 Analyzer), and nuclear magnetic resonance spectroscopy (NMR; Bruker Avance 300) techniques were used for the structural characterization of the acquired epoxy-functional PEG structure. The FTIR spectra of the samples were recorded in the wavenumber range of 400–4000 cm^–1^. ^1^H NMR and ^13^C NMR analyses were performed at 300 and 75 MHz, respectively. The structural and morphological characterizations of the hydrogels from PEG-600-DGE were carried out using FTIR and scanning electron microscopy (SEM; Leo-Evo 40 XVP), respectively. Their thermal features were determined by thermogravimetric analysis (TGA; Shimadzu 50) and differential scanning calorimetry (DSC; Shimadzu 60) analyses. TGA measurements of the samples were carried out from 25 to 800 °C under an air atmosphere at a heating rate of 10 °C min^–1^. DSC analyses were performed from 25 to 500 °C under a nitrogen atmosphere at a heating rate of 5 °C min^–1^.

### 2.4 Determination of Swelling Ratios and Injectability of the Hydrogels

Before swelling studies, hydrogels were dialyzed and dried using a freeze-dryer. Swelling studies were performed in pH 7.4 phosphate buffered saline (PBS) at 37 °C. Dry hydrogels were weighed as *W*_d_, immersed in PBS, and were allowed to reach equilibrium for 48 h. At certain time intervals, excess water on the swollen hydrogels’ surface was eliminated by filter paper, and the hydrogels were weighed (*W*_s_). The percentage swelling of hydrogels was calculated according to eq 1.^19^
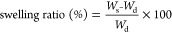
1

For the hydrogels’ injectability measurement, the samples taken in certain amounts were allowed to swell in PBS. 18, 20, 22, 24, and 26 gauge needles were used during the measurement. Their injections were performed by filling the swollen hydrogels into these syringe needles.^37^ Depending on whether the hydrogels flowed from the needle or not, the most suitable gauge needle for injection was determined. The injectability test was carried out in PBS (pH 7.4) at 37 °C. Therefore, different gauge sizes were used to select the most suitable needle. While the thickest needle used is 18 gauge, the thinnest needle is 26 gauge.

### 2.5 Determination of *In Vitro* Biodegradability, Drug Loading, Drug Release, and *In Vitro* Antioxidant Properties of the Hydrogels

In the study, first, 0.1 g of the hydrogel samples were used for *in vitro* biodegradability studies. The samples were incubated in 50 mM pH = 7.4 PBS buffer at 37 °C. The samples were removed at certain time intervals, dried, and weighed. Biodegradability levels were calculated from % lost mass amounts.

Before the hydrogels’ drug loading and release studies were carried out, solutions containing different concentrations of melatonin and β-estradiol were prepared, and a calibration study was performed. The hydrogels placed in dialysis bags were separately immersed in an ethanol-PBS (1:10, v/v) solution containing 0.25 mg mL^–1^ melatonin, ethanol-PBS-tween 80 (0.95:0.05:10, v/v) solution containing 0.1 mg mL^–1^ β-estradiol, and 0.25/0.1 mg mL^–1^ melatonin/β-estradiol solution at 37 °C. After 24 h, the hydrogels were removed from the loading solution. Their surface was rinsed with distilled water and dried.

The hydrogels containing melatonin were left in 20 mL of ethanol-PBS solution at 37 °C for drug release studies. The samples were withdrawn from the release medium at certain times, and the absorbance of melatonin in the medium was spectrometrically measured. Each withdrawn sample was replenished with the same amount of fresh blank solution. The amount of melatonin released was calculated with the help of the calibration curve. The same procedures were repeated for the release solution of β-estradiol, and the amount of released β-estradiol was calculated.^38^ Absorbance values of melatonin and β-estradiol were determined by reading by UV–vis spectrophotometer (UV-1601 Shimadzu, Japan) at 278 and 280 nm wavelengths, respectively.^39,40^

The *in vitro* antioxidant properties with the free radical scavenging effect of a melatonin-loaded hydrogel depending on its release were investigated by 2,2′-diphenyl-1-picrylhydrazyl (DPPH) assay. For melatonin-loaded hydrogel samples, the release was performed in ethanol–water (1:10, v/v) solution under stirring at +4 °C, and samples were withdrawn from the release solution at regular intervals (0, 10, 30, 60, and 180 min). 16 mg of DPPH was dissolved in 100 mL of methanol. Samples were added to the DPPH solution, and the absorbance of the mixture was determined by reading against the blank at 517 nm.^41^

### 2.6 Determination of *In Vitro* Biocompatibility of the Hydrogels

According to ISO-10993-5 (Biological Evaluation of Medical Devices) standards, indirect cytotoxicity of the hydrogel samples on mouse fibroblast cells (L929) was determined with the MTT assay. First, the hydrogels were washed with sterile PBS (pH 7.4) and exposed to UV light for 1 h to ensure sterility. DMEM was added to the samples and was incubated under 5% CO_2_ at 37 °C for 72 h. During incubation, fibroblast cells (5 × 10^4^ cells/mL) were seeded in 96-well plates and kept in an incubator containing 5% CO_2_ at 37 °C overnight. After the incubation, the medium was removed from the hydrogels, and the sample extracts were pipetted into the wells as 100 μL/well. Cells in the control group were treated with the medium only. Plates were incubated in an oven for 24 h. After incubation, the sample extracts were removed from the wells, and fresh medium containing 10% MTT (5 mg mL^–1^, PBS) solution was added. Plates were incubated in the dark for 4 h. Then, the liquid part was removed from the wells, and 100 μL of DMSO was pipetted into each well. The absorbance value in the wells was measured at 540 nm using a microplate reader (Biotek, USA). The data were expressed as a percentage of cell viability, and the absorbances from the control wells were considered 100%.

### 2.7 Asherman’s Syndrome Model

Experimental animal studies of the hydrogels were performed at İnönü University Faculty of Medicine, Experimental Animal Production and Research Center with the permission of the “İnönü University Animal Experiments Local Ethics Committee” and the number of 2015/A-27. Adult female Wistar Albino rats aged 9 months (250–300 g) were used in the experimental modeling. Rats were kept at a constant room temperature of 22 °C in a 12 h light and 12 h dark cycle. Rats were fed standard rat chow and tap water throughout the experimental period without restriction. The menstrual cycles of all rats were synchronized by taking vaginal smears. The animals were divided into 6 groups (*n* = 8), and the following procedures were applied to the animals in the groups. In Group 1 (SHAM), the anterior abdominal wall and uterus of the rat were opened by incision but closed without creating the Asherman model. In Group 2 (ASH), in addition to the procedures in Group 1, the Asherman model was created. In Group 3 (ASH + HDJ), in addition to the procedures in Group 2, the hydrogel was injected into the uterus of the animals. In Group 4 (ASH + HDJ + MEL), in addition to the procedures in Group 2, the melatonin-loaded hydrogel was injected into the uterus of the animals. In Group 5 (ASH + HDJ + EST), in addition to the procedures in Group 2, the β-estradiol-loaded hydrogel was injected into the uterus of the animals and in Group 6 (ASH + HDJ + MEL + EST), in addition to the procedures in Group 2, melatonin + β-estradiol-loaded hydrogel injection was performed into the uterus of the animals.

Drug-loaded hydrogels were adjusted to contain 10 mg/kg melatonin and 0.1 mg/kg β-estradiol daily. The rats were prepared for the operation under ketamine/xylazine anesthesia (50 mg/kg) after the anterior abdominal wall was shaved and stained with iodophor solution. Afterward, a vertical incision of approximately 40 mm was made in the anterior abdominal wall, midline, and the uterus was seen. In order to create the Asherman model, an 18 gauge needle tip was separately inserted to the right and left uterine horns from the cervix to the fallopian tube. A wound was created by moving up and down. Wound formation in the uterine horns in this model system was tested in a pilot study and with histological findings. In the study, Group 2 was not treated, while Groups 3, 4, 5, and 6 were treated by applying approximately 0.5 mL of the hydrogel (no-drug loaded, melatonin, β-estradiol, and melatonin + β-estradiol) to each horn (Figure 2A). The experiment was terminated under sodium pentobarbital (50 mg/kg; intraperitoneal) anesthesia on the 14th day following the formation of the Asherman model, and uterine horns were harvested. Histological and biochemical analyses were carried out on the tissue samples.

#### 2.7.1 Histological Analysis

The uterine horn tissue samples taken from the animals were fixed with 10% formaldehyde, followed by routine histological procedures and embedded in paraffin. Sections of 5 μm thick were taken from paraffin blocks, and tissue sections were stained using hematoxylin-eosin (H-E), periodic acid shiff (PAS), Masson trichrome (MT) staining methods, and the preparations were examined with a microscope and photographed with an image analysis system. Histopathological damage parameters of the uterine horn (epithelial damage, epithelial desquamation, congestion, endometrial thickness, inflammation, and fibrosis) were scored between 0 and 3 and assessed in 10 randomly selected areas (×40 magnification) for each preparation. Accordingly, score 0, score 1, score 2, and score 3 were classified as none (normal), mild (0–25% damage), moderate (25–75% damage), and 3: severe (>75% damage), respectively. All samples prepared for histological measurements were examined and photographed using a Leica DFC-280 light microscope and Leica Q Win Image Analysis System (Leica Microsystems Imaging Solutions Ltd., Cambridge, UK).

#### 2.7.2 Immunohistochemical Evaluation

First, uterine tissue samples taken from animals were routinely processed and embedded in paraffin. Sections of 5 μm thick were cut from paraffin blocks and stained with *K*_i_-67, vascular endothelial growth factor, and HOXA-10 antibodies. Preparations were examined and photographed with a Leica 280 light microscope and the Leica Q Win Image Analysis System (Leica Microsystems Imaging Solutions, Cambridge, UK). In evaluating the immunohistochemical staining results, a semiquantitative general evaluation was made by looking at the staining intensity of surface epithelial and glandular epithelial cells and stromal and vascular endothelial cells in three randomly selected regions at 40× objective magnification. The intensity score was determined as no staining: 0, weak: 1, medium: 2, strong: 3. Data were summarized with median, minimum, and maximum values. The Kruskal–Wallis test was used for group comparisons, and then the Conover method was used for pairwise comparisons. The significance level was accepted as 0.05 in all tests.

#### 2.7.3 Biochemical Analysis

After homogenization and sonification of tissues collected from the uterine horns of the animals under appropriate conditions, supernatants were taken. Then, in the tissue samples, activities of myeloperoxidase (MPO),^42^ catalase (CAT),^43^ superoxide dismutase (SOD)^44^ enzymes, and total glutathione, (tGSH),^45^ nitric oxide (NO), and MDA (lipid peroxidation)^46^ levels were biochemically determined.

### 2.8 Statistical Analysis

Data for biochemical studies and the *in vitro* biocompatibility test were expressed as mean ± standard error. Statistical analyses were performed with GraphPad 8 program using One-Way ANOVA test, and *p* < 0.05 was statistically considered significant. Histological scoring data were expressed as median (minimum–maximum) values. In comparison, the Kruskal–Wallis test and then the Conover pairwise comparison method were used. Data were summarized with the mean ± standard deviation, and One-Way analysis of variance was used for comparison. The significance level was accepted as 0.05 in all tests.

## 3 Results and Discussion

Hydrogels were preferred in our study because of their key characteristics for its usage in Asherman’s syndrome such as biocompatibility, hydrophilicity, and slow drug release. In the hydrogel structure, β-CD as a cross-linker, PEG as a biocompatible and swellable polymer, and PPhs as oxidative stress inhibitors were used. These hydrogels contain also β-estradiol and melatonin that can eliminate oxidative stress in the uterus, improve uterine damage, and prevent intrauterine adhesions. There is only one study in the literature on CD-based hydrogels, and it has aimed to treat cervical inflammation using hydroxypropyl-γ-CD hydrogels.^47^ No studies were found to prevent or treat Asherman’s syndrome using CD-based hydrogels. There are limited studies involving hydrogels to prevent or reduce Asherman’s syndrome in the current literature. In these studies, attempts were made to prevent adhesions using hyaluronic acid–based hydrogels,^48−53^ methacrylated gelatin, methacrylated collagen composite hydrogels,^54^ and available spray gel kits.^55^ However, drug-loaded CD-based hydrogels are also not available in the literature to prevent uterine damage and Asherman’s syndrome. It has been considered that these hydrogels containing melatonin^56^ and β-estradiol^57^ to improve uterine damage, prevent intrauterine adhesions, and eliminate oxidative stress in the uterus as a result of damage may offer a significant advantage.

In the study, while designing the structure of hydrogels, polar, linear, and water-soluble structures were preferred as monomers to contribute to hydrophilicity. In the synthesis, CDs were used to improve the drug carrier property by host–guest interaction and to form a network structure with multiple bindings. Due to biocompatibility, PPhs were utilized to increase the mechanical barrier property of the hydrogel after degradation, while the PEG structure was used to ensure intrabody safety and flexibility. Their cross-linking and PPh ratios were kept low to contribute the hydrogels to intrabody flexibility. In order to be used easily in intrauterine applications, hydrogel structures with high biocompatibility have been prepared not to create a feeling of foreign body and obstruction in the applied structure. CD-based hydrogels using epoxy-functional PEG and different PPhs were synthesized, and their structural, thermal, morphological, physical, biological, histological, and biochemical properties were investigated using *in vitro* and *in vivo* studies. The structural and thermal characterizations of the epoxy-functional PEG and hydrogels are provided in the Supporting Information.

### 3.1 Structural Characterization of Poly(ethylene glycol) 600 Diglycidyl Ether Structure

**Figure 3:**
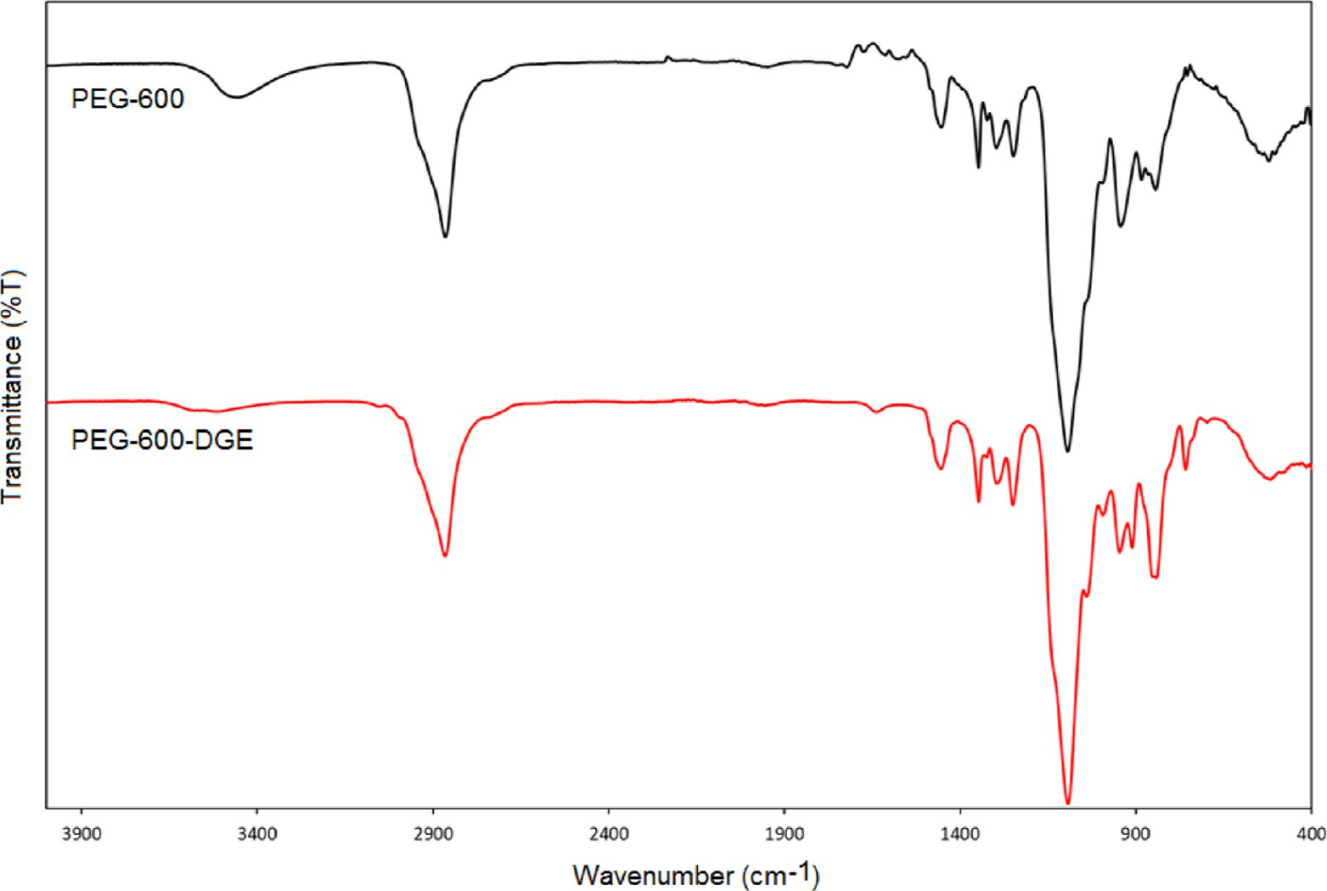
FTIR spectra of PEG-600 and PEG-600-DGE structures.

**Figure 4:**
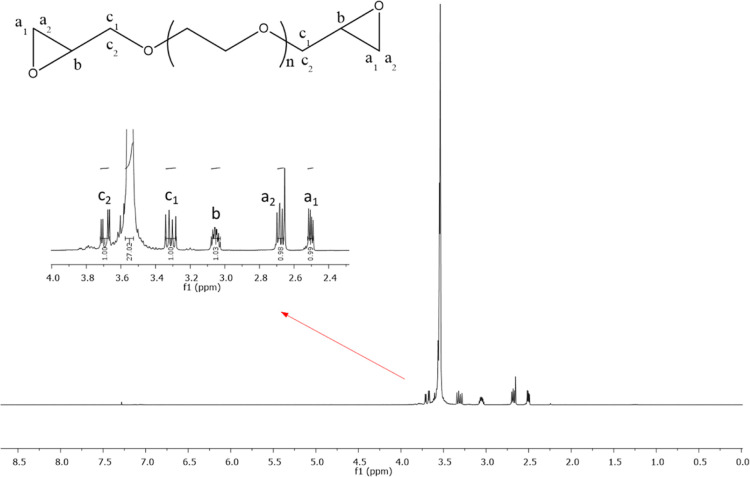
^1^H NMR spectra of PEG-600-DGE.

**Figure 5:**
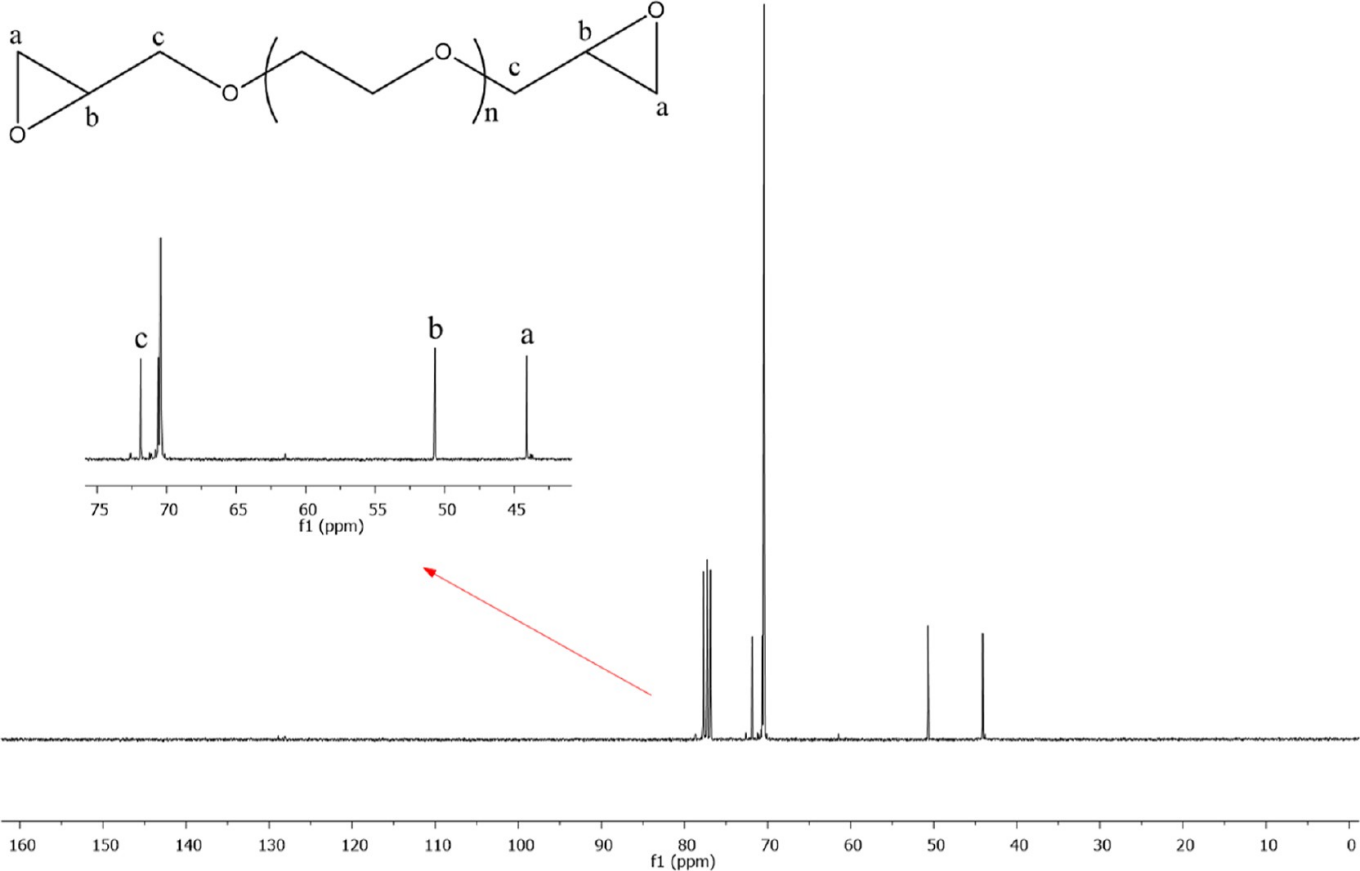
^13^C NMR spectra of PEG-600-DGE.

Before the synthesis of the hydrogels, the poly(ethylene glycol) 600 diglycidyl ether (PEG-600-DGE) structure from poly(ethylene glycol) 600 (PEG-600) and epichlorohydrin was confirmed using FTIR, ^1^H NMR, and ^13^C NMR, and the results are presented in Figures 3–5, respectively. According to the FTIR spectra (Figure 3), the characteristic epoxide absorption peaks that are not found at PEG-600 were seen as asymmetrical ring bending at 910 cm^–1^ and C–H bending at 759 cm^–1^ at PEG-600-DGE.^34,35^ In addition, the epoxide peaks belonging to PEG-600-DGE were symmetrical ring stretching at 1252 cm^–1^, C–H stretching at 2997 cm^–1^, and CH_2_ stretching at 3052 cm^–1^. These peaks explained the binding of epichlorohydrin and proved that the epoxy ring was closed. In addition, it was seen that the broad hydroxyl peak around 3436 cm^–1^ belonging to PEG-600 disappeared after the formation of PEG-600-DGE. The disappearance of this peak was another proof that the ring is closed. The ^1^H NMR and ^13^C NMR data of the obtained epoxy-functional PEG are given in Figures 4 and 5.

The ^1^H NMR and ^13^C NMR spectra of the obtained epoxy-functional PEG structures are given (Figures 4 and 5). According to the ^1^H NMR spectra, methylene and methine groups in the PEG-600-DGE structure showed similar chemical shifts. The characteristic peaks at 3.67–3.71 (m, 2H) and 3.28–3.34 (m, 2H) ppm are thought to belong to methylene hydrogen (c_2_ and c_1_). The peaks at 3.03–3.08 (m, 2H) ppm were the methine hydrogen signal (b). The peaks at 2.65–2.70 (t, 2H) and 2.49–2.52 (m, 2H) ppm were due to methylene hydrogen (a_2_ and a_1_) (Figure 4). These results confirm that epichlorohydrin is chemically attached to both ends of the PEG chain.^34^

In the ^13^C NMR spectra, characteristic peaks in the PEG-600-DGE structure are observed. In the ^13^C NMR results, after the epichlorohydrin binding to PEG, distinct peaks belonging to C atoms in the epoxy ring are observed at 44.10 ppm (a) and 50.70 ppm (b) (Figure 5).^35,36^

The PEG-600-DGE structure was obtained using a 2:1 ratio of epichlorohydrin/PEG. In this way, hydrogel formation was achieved at lower temperatures thanks to the epoxy-containing PEG structure. In addition, the PPhs desired to be incorporated into the hydrogel structure have bounded by covalent bonds without decomposition. The synthesis of cyclodextrin-based hydrogels was performed using epoxy-functional PEG, β-CD, and different PPhs.

### 3.2 Structural and Thermal Characterization of the Hydrogels

**Figure 6:**
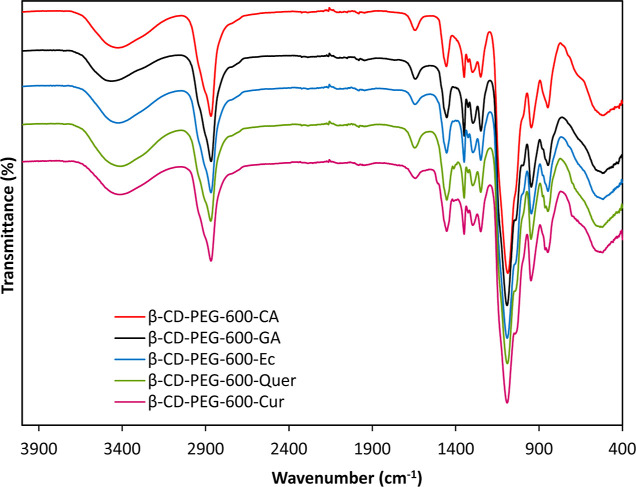
FTIR spectra of β-CD-PEG-600 hydrogels from different PPhs.

**Figure 7:**
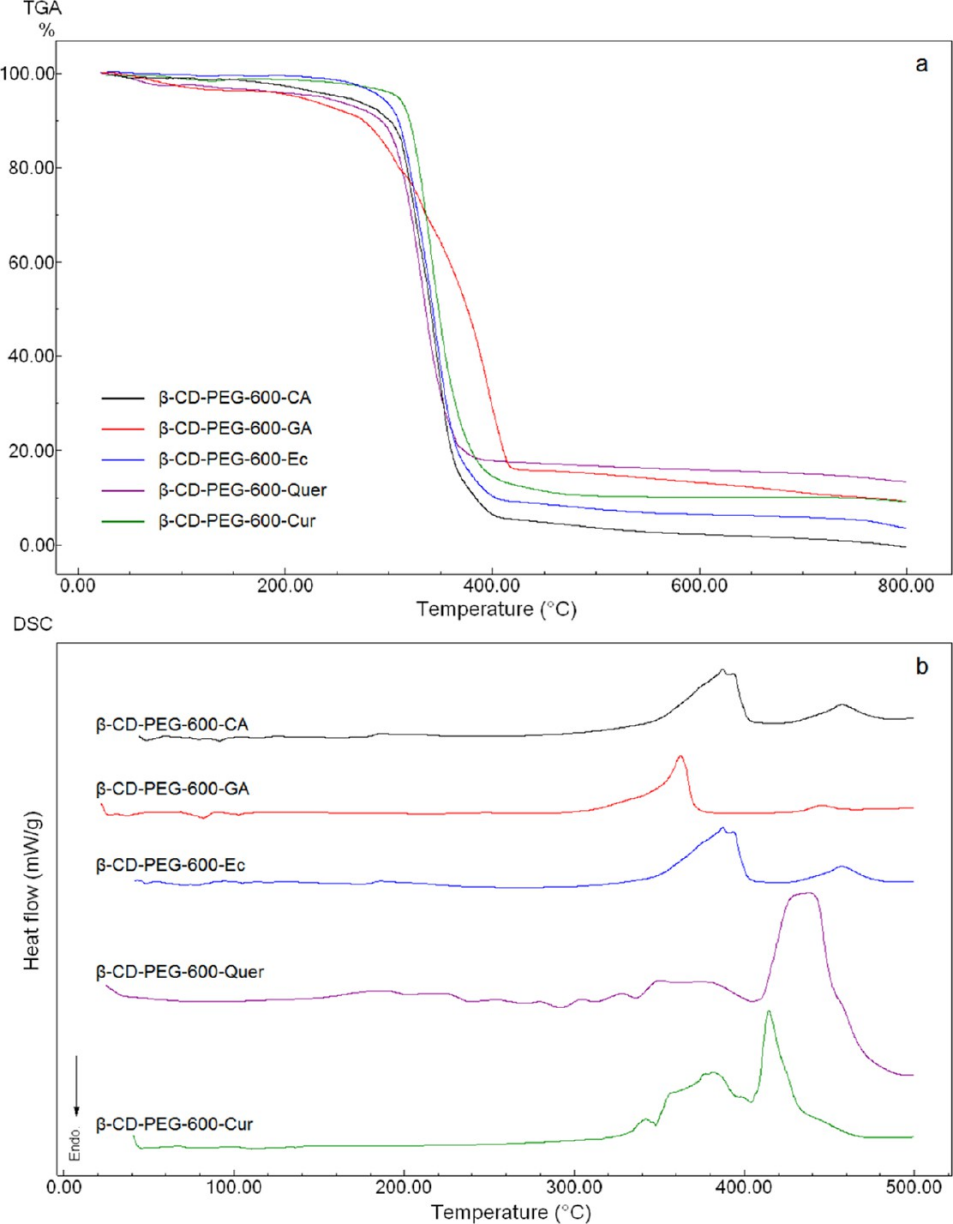
TGA (a) and DSC (b) thermograms of β-CD-PEG-600 hydrogels from different PPhs.

Hydrogel structures were synthesized using the synthesized PEG-600-DGE structures and the monomer ratios in Table 1. Their reaction yields were generally high, and stability increased with the increasing cross-linking rate. First, the resulting hydrogels were characterized by FTIR and thermal analysis methods (TGA and DSC). The spectra of these characterization are given in Figures 6 and 7, respectively. The success of the polymerization was followed by FTIR spectroscopy. According to the spectra, depending on the PEG groups in the hydrogel structure, the aliphatic C–H stretching at 2840–3050 cm^–1^, C–O–C etheric stretching at 1170 cm^–1^, and C–O stretching vibrations at 1050 cm^–1^ were observed. The aliphatic C–H shoulder peak at 2950 cm^–1^, free OH peak at 1650 cm^–1^, C–H stretching peak at 1465 cm^–1^, and C–O–C peak at 1413 cm^–1^ originating from the β-CD were observed. Peaks belonging to PPhs were weaker because their ratio is less than PEG. However, when the spectra were examined in detail, C–H and C=C peaks originating from aromatic units were observed at 825 and 1505 cm^–1^, respectively. In addition, it was seen that the C–H bending peak at 759 cm^–1^ and the asymmetrical ring bending peak at 910 cm^–1^, which were the prominent peaks of the epoxy ring, disappeared entirely in the spectra. These peaks showed that the desired hydrogel structure was formed by opening the ring. A large part of the hydrogel structure consisted of PEG units in the hydrogels. The ratio of PEG during synthesis was around 90%, so the change in the FTIR spectra were not very clear. However, the swelling and elastic structure of the product proved that the hydrogel was formed. Since most of the hydrogel structures were structurally composed of PEG, peaks belonging to the –CH_2_–CH_2_–O–CH_2_ sequence were generally seen in the FTIR spectra of hydrogels.

The thermal properties of β-CD-PEG-600 hydrogels with different PPhs were examined with TGA and DSC analyses as seen in Figure 7a,b. According to the thermal analysis results, it can be stated that the thermal stability of all β-CD-PEG-600 hydrogels was around 300 °C. When the TGA thermograms of the obtained hydrogels are examined, the TGA thermograms of other hydrogels, except β-CD-PEG-600-GA, are seen to be similar. However, two different mass losses were observed in β-CD-PEG-600-GA and β-CD-PEG-600-Quer hydrogels. The first mass loss is the mass loss caused by the removal of moisture in the structure between 80 and 130 °C. The second mass loss is due to the degradation of the hydrogel structure. In β-CD-PEG-600-CA, β-CD-PEG-600-Cur, and β-CD-PEG-600-Ec hydrogel structures, a basic mass loss is observed, which starts at approximately 300 °C and completes at 420 °C. Although the polyphenol groups bound to the structure are low in proportion, they affect the thermal properties of the structures because polyphenol groups change the cross-linking rates and affect the pore structure, mechanical properties, and thermal stability of polymers. The exotherm resulting from the degradation of the hydrogel structure seen in the DSC curves of the hydrogel structures is between approximately 310 and 400 °C. After 400 °C, another exotherm due to carbonization is observed. In the DSC curves for β-CD-PEG-600-CA, β-CD-PEG-600-GA, β-CD-PEG-600-Ec, β-CD-PEG-600-Quer, and β-CD-PEG-600-Cur, degradation onset temperatures are around 320, 330, 370, 340, and 325 °C, respectively. The reason for this is that the stability of the structure increases depending on the cross-linking rate in the hydrogel structure. Since GA, Ec, and Quer polyphenol structures carry more than two – OH groups, they increased the amount of cross-linking and branching. Therefore, hydrogel structures prepared with these PPhs (GA, Ec, and Quer) are structurally more stable.

### 3.3 Determination of Swelling Properties, Injectability, and Morphological Structure of the Hydrogels

**Figure 8:**
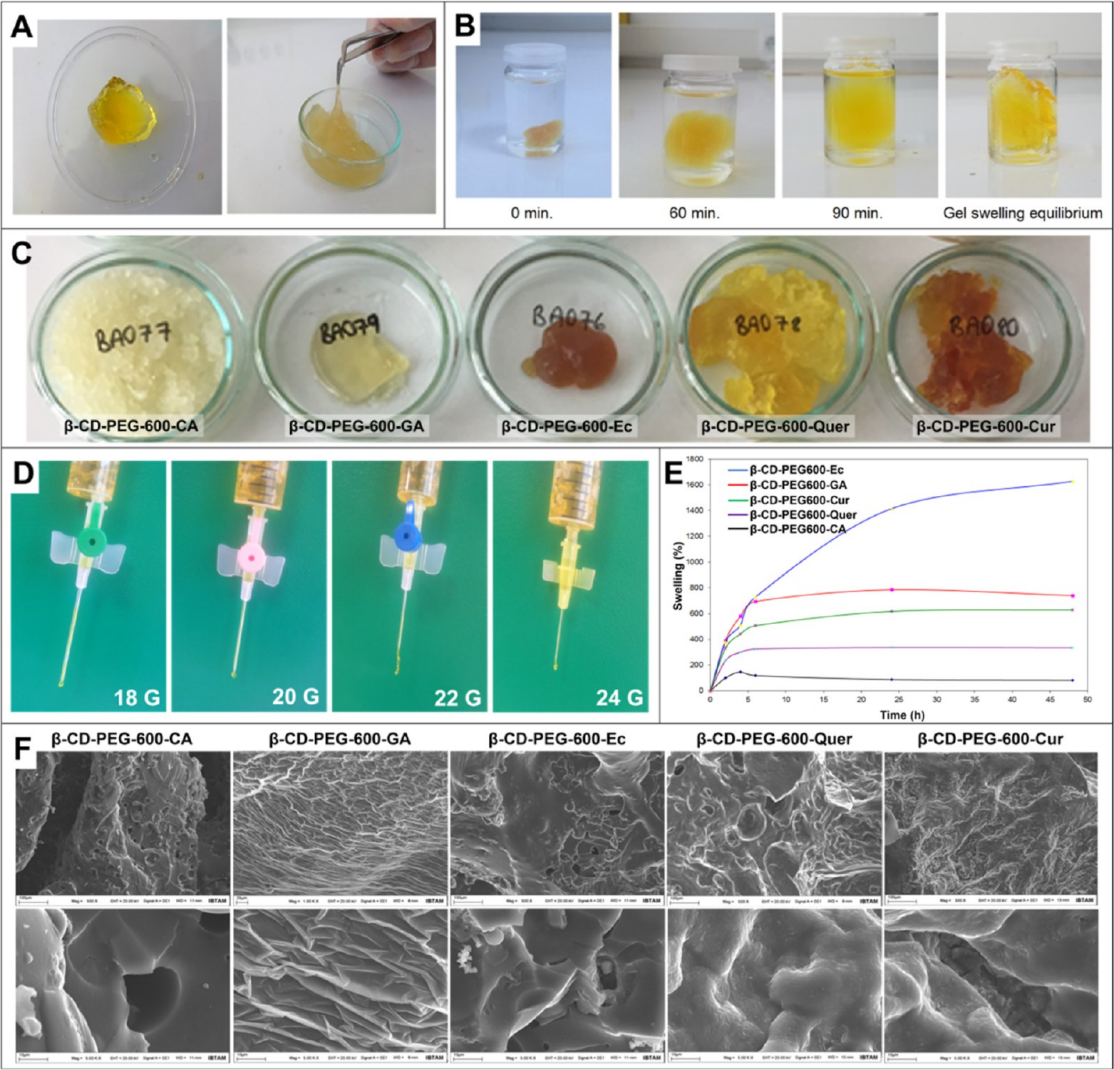
(A) General view of the hydrogels. (B) Time-dependent swelling images of β-CD-containing (β-CD-PEG-600-Quer) hydrogels. (C) General views of β-CD-PEG-600-based hydrogels. (D) Injectability test images of hydrogel structures (β-CD-PEG-600-Cur) for 18, 20, 22, and 24 gauge needles. (E) % Swelling test results of β-CD-PEG-600 hydrogels from different PPhs. (F) SEM images of β-CD-PEG-600 hydrogels from the different PPhs.

One of the most critical stages of the study was the control of the successful synthesis of the hydrogels. Their general appearance was as in Figure 8A–C. The synthesized hydrogels were obtained in a flexible, homogeneous, colorful, and rapidly swelling form.

The basic monomeric units, PEG and , were colorless, and the colored structures of the hydrogels were due to the various PPhs. Since the colors of the hydrogels are not lost during washing, swelling, and release stages, we understood that the PPhs are covalently bound to the hydrogel structure. Hydrogel structures prepared using only PEG and β-CD were light yellow, while hydrogels prepared with PPhs were light yellow, yellow, or orange. Since PEG has biocompatibility and high flexibility, it is widely used in many biomedical applications and drug designs today. For this reason, the PEG ratio was kept high in the hydrogel synthesis. Furthermore, since the ring-opening reaction occurs at low temperature, it was achieved to bind the PPhs to the hydrogel structure without degradation. In the study, five hydrogel structures were prepared using epoxy-functional PEG-600, β-CD, and different PPhs. The structural difference has been provided by diversifying these structures to include CA, GA, Ec, Quer, and Cur. Hydrogel structures obtained using β-CD, PEG-600-DGE, and different PPhs are given in Figure 8C. These hydrogels were bright, homogeneous, highly flexible, durable, and swellable. When they swelled, their transparent appearances increased. They swelled and shrunk as one piece. Their colors vary depending on the PPhs.

The aim of the study is to synthesize, characterize, and determine *in vivo* efficiency of cyclodextrin-based hydrogels at different pore sizes and cross-linking ratios, which can be used in postintrauterine surgical interventions and reconstitution of damaged uterine tissue to prevent possible intrauterine adhesions. Therefore, cyclodextrin-based injectable hydrogels (Figure 8A) with mechanical barrier properties have been synthesized to prevent the formation of Asherman’s syndrome with this study. The structures of these hydrogels are composed of PEG, PPhs, and cyclodextrin groups as cross-linkers. Figure 8 shows the general structures, swelling test results, injectability test images, and SEM images of these synthesized hydrogels. In Figure 8A, it can be seen that the synthesized hydrogel structure is flexible in the swollen state. In addition, it is clearly seen that this swelling process is quite rapid and reaches the equilibrium swelling structure in approximately 90 min (Figure 8B). In Figure 8C images, it was seen that the hydrogels swell homogeneously, and this high swelling behavior proved the hydrogel structure of the material. These high swelling of hydrogels is connected with the hydrophilicity of their functional groups and the low cross-linking degree. The color of the hydrogels originated from chromophore units of the PPhs. Furthermore, the absence of any color during swelling in the solution also indicated that the PPhs were covalently bound to the hydrogels.

Hydrogels should be in an injectable form for application inside the body. The thin injector tip is important for the patient to feel less pain during the application. Therefore, 18, 20, 22, 24, and 26 gauge needle tips were used to determine the injectability of the hydrogels as seen in Figure 8D. Injectability test results of the hydrogels are shown in Table S1 (the thickest needle is 18 gauge, while the thinnest needle is 26 gauge). According to these results, the injectability of the hydrogels was high. It was determined that GA-, Ec-, Quer-, Cur-based hydrogels were injectable in the 18, 20, 22, and 24 gauge injectors, and Ec-, Quer-, Cur-based hydrogels were injectable in the 26 gauge injector. The swelling graphs of the hydrogels are shown in Figure 8E. According to the swelling test results, it was seen that the swelling capacity of the hydrogels was high, and most of them reached equilibrium swelling values in about 5–6 h, depending on their structure. Especially β-CD-PEG-600-Ec has the highest swelling capacity with a value of 1600%, while β-CD-PEG-600-GA, β-CD-PEG-600-Cur, β-CD-PEG-600-Quer, and β -CD-PEG-600-CA had a swelling rate of 700, 550, 300, and 100%, respectively. Feng *et al.* reported the swelling capacity of the 3D printed GelMA/ColMA hydrogel as 19%,^54^ and Wenbo *et al.*(26) reported the swelling capacity of the chitosan-heparin hydrogel with SDF-1α controlled release manner for intrauterine antiadhesion.^33^ Wenbo *et al.*(26) showed that SDF-1α-controlled release chitosan-heparin hydrogels were effective after a 7-day treatment in rats with uterine damage.^33^

Obtained hydrogel structures have two different pores in their structure. These are porosity resulting from β-CD groups and porosity resulting from cross-linking. In the study, porosity resulting from β-CD groups serves to transport melatonin groups through host–guest interaction. The large pore structure formed by cross-linking enables the absorption of β-estradiol structures. In this way, the hydrogel structures obtained release β-estradiol and melatonin in the uterus, allowing the damaged uterus and uterine horns to heal faster.

The resulting hydrogel structures were lyophilized while swollen and their pore structures were examined by SEM analysis (Figure 8F). In addition, morphological characteristics of hydrogels were investigated by SEM, and their low and high magnification images are illustrated in Figure 8F. From the images, it was observed that the surfaces of the lyophilized hydrogels were not smooth. Porous and cavity structures and distinct holes were observed. Besides, the hydrogels’ homogeneity, surface cavities, and pore structures were seen. These images were similar to the morphologies of hydrogels available in the literature. The high swelling ability of hydrogels could be due to their porous structures.

### 3.4 Determination of *In Vitro* Biodegradability, Drug Release, and *In Vitro* Antioxidant Properties of the Hydrogels

**Figure 9:**
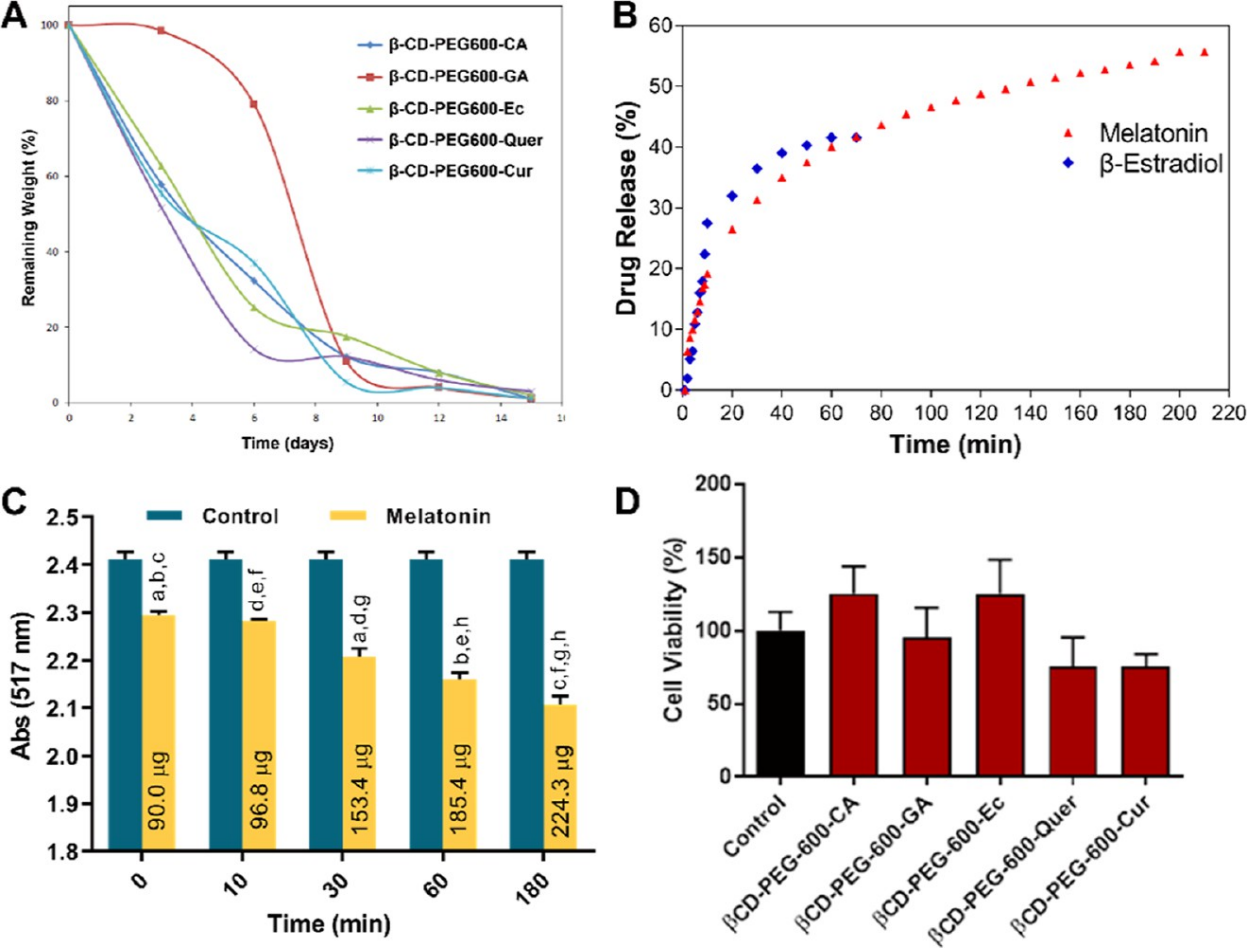
(A) *In vitro* biodegradability results of β-CD-PEG-600 hydrogels from different PPhs. (B) Melatonin and β-Estradiol release curve of the β-CD-PEG-600-Ec hydrogel. (C) Antioxidant activity of the β-CD-PEG-600-Ec hydrogel depending on the result of melatonin release; a; 0–30 min, b; 0–60 min, c; 0–180 min, d; 10–30 min, e; 10–60 min, f; 10–180 min, g; 30–180 min, h; 60–180 min; *p* < 0.05. (D) Cell viability of β-CD-PEG-600 hydrogels from the different PPhs on L929 cells.

In the *in vitro* biodegradability test, it was determined that all hydrogels were degraded by approximately 80–90% at the end of the 9-day period (Figure 9A). The main purpose of the hydrogels synthesized in the study is to prevent adhesions that may occur due to scar tissues formed during the healing process after intrauterine damage. For this reason, the synthesized gels are primarily gels with mechanical barrier properties. They prevent the damaged uterine walls from adhering. In intrabody wounds, the healing time of the wound is generally determined as 4–7 days. For this reason, the gel must remain in the area where it is applied throughout this period. Therefore, the synthesized hydrogel must remain in the application area for at least 8–9 days. The second purpose of the synthesized hydrogels is to ensure faster healing of wounds and damage in the uterus. For this reason, drug loadings have been made to accelerate wound healing. In order to quickly trigger wound healing, the hydrogel structure was designed to rapidly release the drug it absorbs. According to the biodegradability results, the hydrogels started to degrade during the wound healing period and showed a peak degradation after this period. On the 15th day, it was seen that the hydrogels were completely degraded and this was an indication of no hydrogel residue left.

Melatonin and β-estradiol release studies of the β-CD-PEG-600-Ec hydrogel, which has the highest biocompatibility, were performed, and the results are shown in Figure 9B. During the release, in the first 10 min, the absorbance of the solution was read every min and then every 10 min. Generally, hydrogels had a rapid release within the first 60 min but then a slower one. β-estradiol released around 40% in 80 min while melatonin released around 55% in 220 min from the hydrogel, and these release levels were sufficient for biological applications. Chen *et al.* found that approximately 30% of β-estradiol was released in 24 h from the human amniotic extracellular matrix scaffold for endometrium regeneration.^58^ Melatonin contributes to uterine healing,^59,60^ but there is no melatonin-loaded hydrogel design in the literature. Torabi *et al.* synthesized thermosensitive melatonin-loaded conductive pluronic/chitosan hydrogel for myocardial tissue engineering and reported that ∼60% of melatonin was released in 70 h.^61^ Xiao *et al.* reported the releasing of melatonin as 85–90% in 5 days from gelatin methacryloyl-dopamine liposomes.^62^

The radical-scavenging power of the melatonin-loaded β-CD-PEG-600-Ec formulation was determined by the DPPH test. In the experiment, it was checked whether the released melatonin preserved its activity at certain time intervals. As seen in Figure 9C, melatonin was released depending on time as 90, 96.8, 153.4, 185.4, and 224.3 μg at 0, 10, 30, 60, and 180 min, respectively. Increasing melatonin level over time was consistent with the release studies, and it also preserved its radical-scavenging effect. The decreases in absorbance at 30th, 60th, and 180th min were significant compared to the zeroth and 10th min (*p* < 0.05). The decrease in absorbance at the 180th minute was found to be significant compared to the 30th and 60th min (*p* < 0.05). An absorbance decrease was observed at the 10th min compared to the zeroth min, but this result was not significant (*p* > 0.05). The significant trend of the decreases also showed that melatonin did not lose the activity due to its release with time.

### 3.5 *In Vitro* Biocompatibility Studies

The cytotoxicity tests of the hydrogels were performed using the indirect method for assessing biocompatibility. According to the results, the cell viability values of the hydrogels are depicted in Figure 9D. In the biocompatibility test on L929 cells, it was observed that the cells exhibited viability of about 125.2, 95.6, 125.9, 75.6, and 75.9% for β-CD-PEG-600-CA, β-CD-PEG-600-GA, β-CD-PEG-600-Ec, β-CD-PEG-600-Quer, and β-CD-PEG-600-Cur, respectively. It was determined that all samples showed cell viability greater than 70% of the untreated control as recommended by the ISO10993-5. *In vitro* biocompatibility results showed that especially β-CD-PEG-600-CA and β-CD-PEG-600-Ec hydrogels’ extract had no toxicity, and they could serve as a safe antiadhesion material.

Since the β-CD-PEG-600-Ec had the highest biocompatibility, highest swelling degree, and injectability in all injectors, this formulation was chosen for the Asherman’s Syndrome rat model.

### 3.6 Application of Hydrogels in the *In Vivo* Asherman Rat Model System

**Figure 10:**
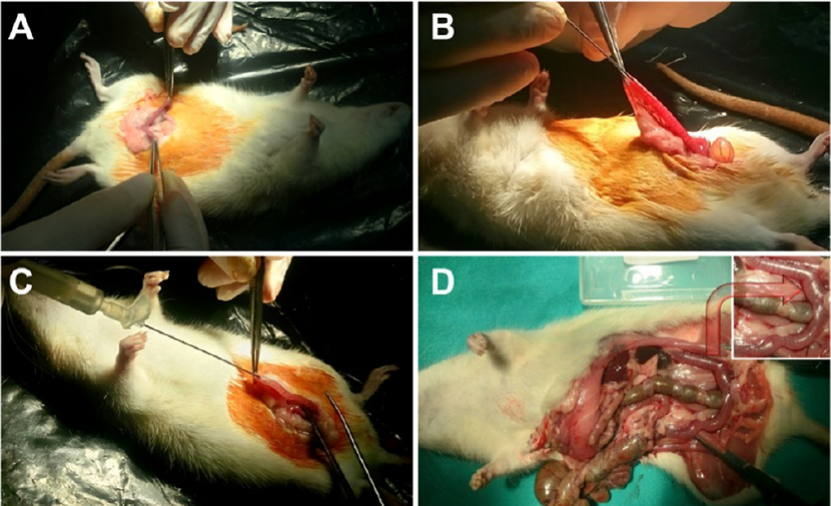
(A) Detection of uterine horns of rats. (B) Injury of the uterine horns with an 18 gauge needle tip to induce Asherman’s syndrome. (C) Hydrogel injection into injured uterine horns. (D) Image of uterine horns of rats after 14 days (*n* = 8).

At this stage of the study, based on *in vitro* experiment results, the β-CD-PEG-600-Ec hydrogel, which has the most optimum properties among the hydrogels, was selected and applied in the Asherman rat model system (Figure 10). During the 14-day application period, the viability ratio of the animals was 100%. After the application, it was determined that uterine horns contained a certain amount of hydrogel, depending on the size (Figure 10D).

### 3.7 Histological Evaluation

H-E, PAS, and MT stainings were performed on tissue samples taken from rat uterine horns after β-CD-PEG-600-Ec hydrogel application. Histopathological parameters such as epithelial damage, epithelial desquamation, congestion, endometrial thickness, inflammation, and fibrosis were evaluated.

#### 3.7.1 Epithelial Damage, Desquamation, and Congestion

**Figure 11:**
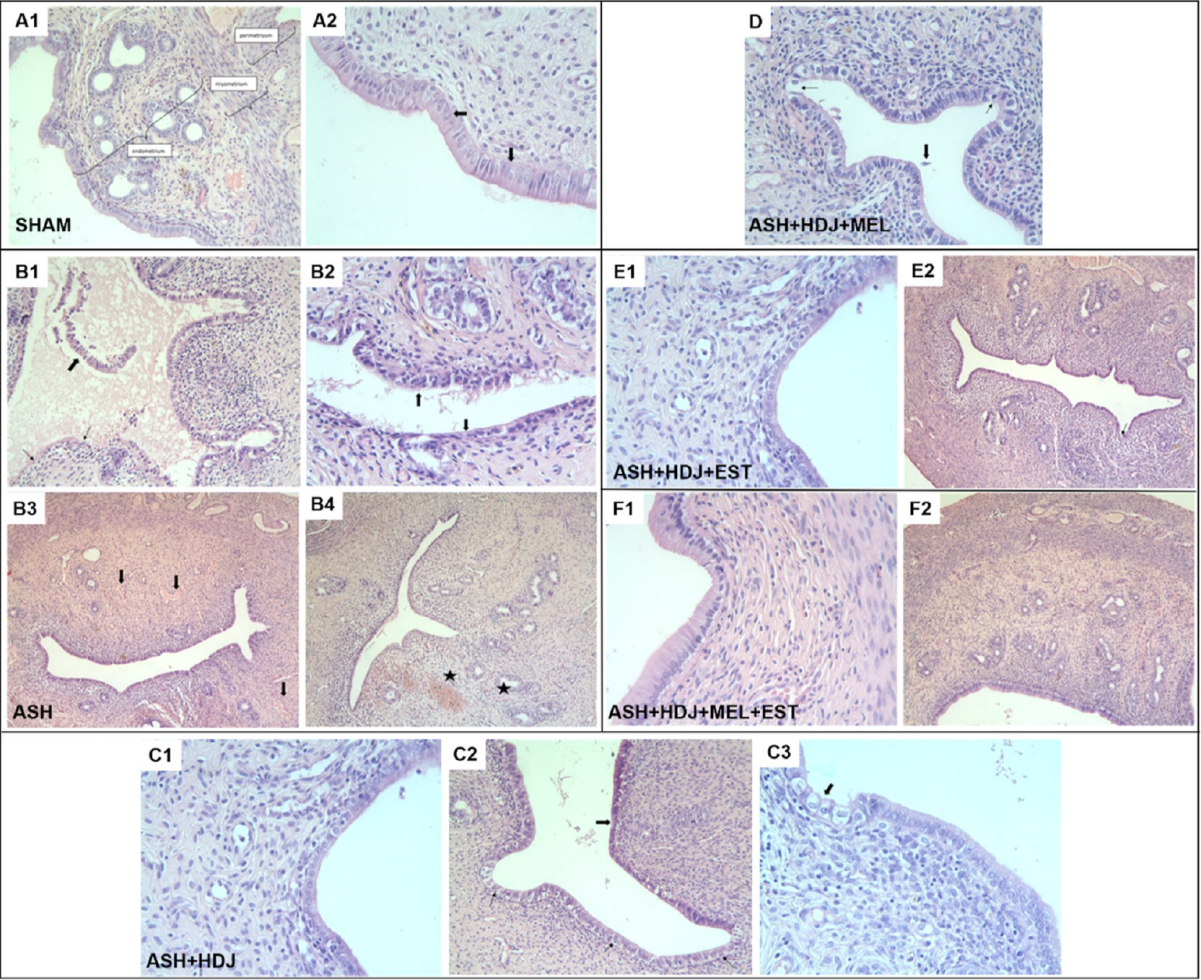
(A) H-E staining of tissues belonging to the SHAM group (A2; H-Ex40, arrow: epithelial nucleus positioned in the same plane). (B) H-E staining of tissues belonging to the ASH group [(B1) thick arrow: severe epithelial desquamation, thin arrow: epithelial sequestration and thinning, (B2) thick arrow: epithelial damage and thinning, (B3) decreased glands and congestion in the endometrial mucosa, arrow; congestion, and (B4) Asterisk; hemorrhagic areas]. (C) H-E staining of tissues belonging to the ASH + HDJ group [(C2) thick arrow: damage and thinning of the epithelium, thin arrow: some small epithelial nuclei and (C3) thick arrow: damaged epithelium]. (D) H-E staining of tissues belonging to the ASH + HDJ + MEL group (thick arrow: exfoliated epithelial cell in the lumen, thin arrow: damaged epithelium). (E) H-E staining of tissues belonging to the ASH + HDJ + EST group [(E2) thin arrow: epithelial thinning].(F) H-E staining of tissues belonging to the ASH + HDJ + MEL + EST group.

**Figure 12:**
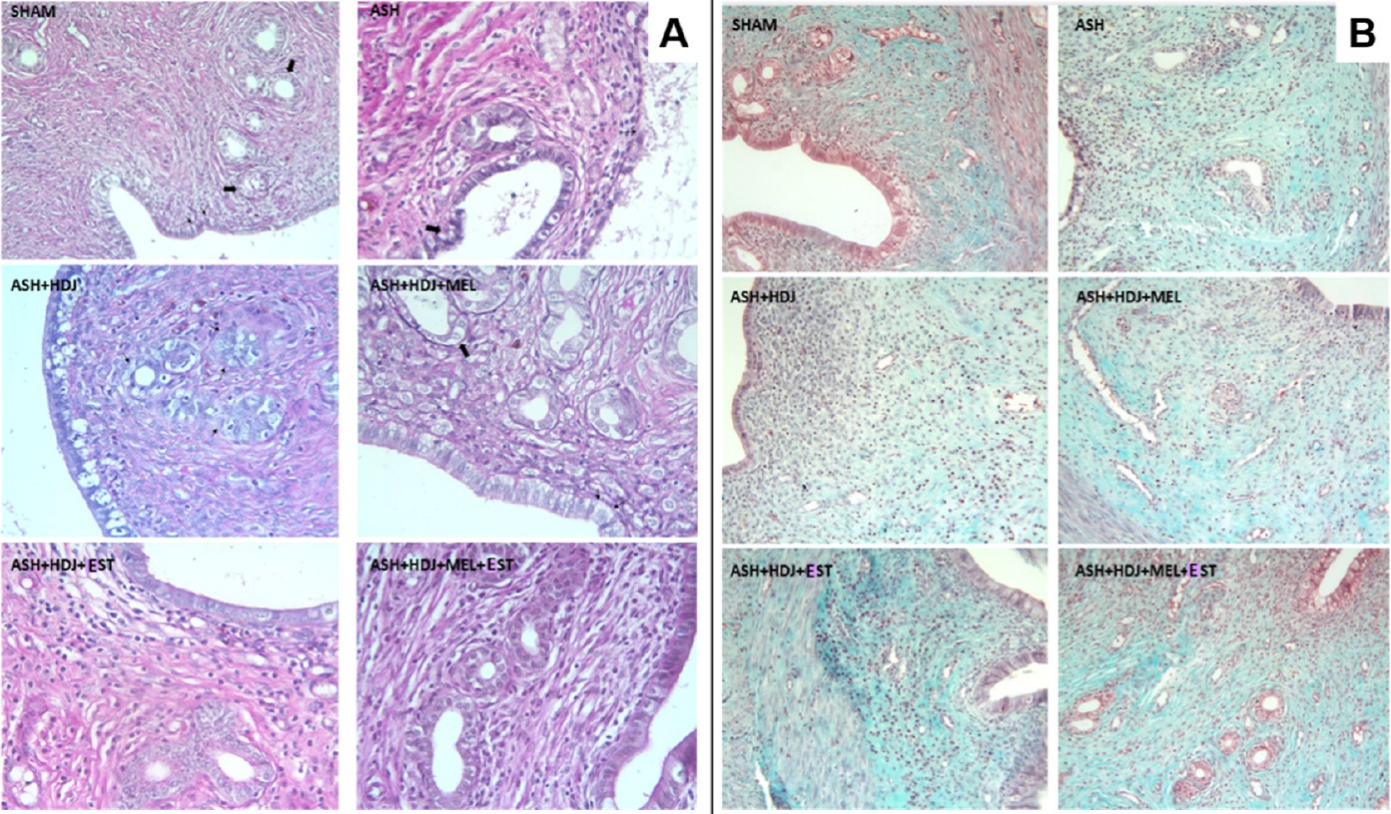
(A) PASx40 staining of tissues (SHAM: thin arrow: epithelial basal membrane, thick arrow: glandular epithelial basal membrane, ASH: thin arrow: desquamation of basal membrane and epithelium, thick arrow: disappeared glandular epithelial basal membrane, ASH + HDJ: arrow: disappeared glandular epithelial basal membrane, ASH + HDJ + MEL: thin arrow: desquamation of endometrial basal membrane, thick arrow: desquamation of gland epithelium) and (B) MTx20 staining of tissues.

The H-E staining method was applied to determine the epithelial damage, desquamation, and congestion, which were the criteria to observe the Asherman’s syndrome created in the experimental model and the effectiveness of the substances given afterward. The PAS staining method was applied to evaluate the basal lamina between the epithelium and the lamina propria. Light microscope examination results are given in Figures 11 and 12A.

In the general view of the SHAM group, the uterine horn sections were in a normal histological structure, the perimetrium, myometrium, and endometrium layers were in order and regular, the lumen was uninterrupted, and glands were in a normal structure. At the same time, congestion, one of our criteria, was not observed in any area. It was observed that the contours of the epithelium covering the surface of the endometrium were straight, the size and shape of the cells, and the nucleus shapes and positions were normal (Figure 11A1,A2). The basal membrane structure, which was examined by PAS staining, and on which rested both the endometrial surface epithelium and the gland epithelium, were uninterrupted (Figure 12A). As a result of the median (min–max) evaluations in the scoring of this group, epithelial damage was 0 (0–1), epithelial desquamation was 0 (0–2), and congestion was 0 (0–2) (Table S2).

In the general view of the ASH group, the perimetrium, myometrium, and endometrium layers were thinned and thickened in some places and exhibited an abnormal appearance, while the lumen was found to be irregular and intermittent due to both epithelial damage-desquamation and degeneration of the layers. Damaged areas on epithelial examination were seen quite commonly. In addition, desquamation in many areas due to damage, stainings in the lumen depending on the desquamation, irregular epithelial borders, degeneration in surface epithelial cells, contour irregularity due to swelling, decrease in their height, flattening-like shape changes in the nuclei, and marked congestion were observed (Figure 11B1,B2). A noticeable decrease in the density of the glands was detected locally (Figure 11B3,B4). It was observed that the basal membrane examined with PAS staining was thinned from place to place, partly separated from the endometrial epithelium, damaged, and interrupted (Figure 12A). The same findings were also detected in the basal membrane of the gland epithelium in sections belonging to this group. These findings demonstrated that damage depending on Asherman’s syndrome could be created in the experimental group. As a result of the median (min–max) evaluations in the scoring of this group, epithelial damage was 3 (2–3), epithelial desquamation was 1 (0–3), and congestion was 2 (1–3) (Table S2).

In the uterine horn sections of the ASH + HDJ group, the damage and desquamation observed in the Asherman group continued. The shrinkage, flattening in nuclei of some surface epithelial cells, contour irregularity in their shapes due to swelling, and a decrease in their height in some cells were observed (Figure 11C1–C3). Generally, it was determined that the severity of epithelial damage, desquamation, and congestion decreased. It was observed that the thinning and irregularities seen in the ASH group in the basal membrane examined by PAS staining and the irregularities in the basal membranes of the gland epithelium decreased (Figure 12A). As a result of the median (min–max) evaluation in the scoring of the ASH + HDJ group, epithelial damage was 2 (1–3), epithelial desquamation was 0 (0–2), and congestion was 1 (0–2) (Table S2).

In the uterine horn sections of the ASH + HDJ + MEL group, perimetrium, myometrium, and endometrium layers were found to be close to normal in general view. The parameters of epithelial damage, desquamation, and congestion were significantly reduced but persisted, although their severity was reduced in some areas (Figure 11D). Furthermore, improvements were observed in shape changes due to degeneration detected in the epithelial cells of the ASH group. In the PAS staining, it was observed that the basal membrane of the glands of the endometrium was thinned in some areas, as in the ASH + HDJ group, but this thinning was not evident as in the ASH group (Figure 12A). As a result of the median (min–max) evaluation in the scoring of ASH + HDJ + MEL group, epithelial damage was 1(0–2), epithelial desquamation was 0 (0–2), and congestion was 1 (0–2) (Table S2).

Although the damage to the endometrial epithelium in the uterine horn sections of the ASH + HDJ + EST group was reduced compared to the ASH group, it was observed that the thinned surface areas and the congestion continued. It was determined that the density of the glands increased in the endometrial mucosa (Figure 11E1,E2). In the PAS staining of the group, basal membrane findings similar to the ASH + HDJ + MEL group were seen (Figure 12A). As a result of the median (min–max) evaluation in the scoring of the group, epithelial damage was 2 (0–2), epithelial desquamation was 0 (0–2), and congestion was 1 (0–2) (Table S2).

When the uterine horn sections of the ASH + HDJ + MEL + EST group were examined, it was determined that all histological parameters of the uterine horn were similar to the SHAM group in H-E staining (Figure 11F1,F2). In the PAS staining of the ASH + HDJ + MEL + EST group, normal histological findings, same as the H-E staining, were observed in the basal membrane structure of the endometrial epithelium and glands (Figure 12A). In the scoring of the group, as a result of the median (min–max) evaluation, epithelial damage was 0 (0–1), epithelial desquamation was 0 (0–2), and congestion was 0 (0–1) (Table S2).

#### 3.7.2 Endometrial Thickness

The H-E staining method was applied to the sections to determine the endometrial thickness. As a result of light microscopy examination of the SHAM group, the uterine horn sections were in the normal histological structure. The perimetrium, myometrium, endometrium layers, and endometrial thickness were normal (Figure 11A1,A2). The endometrial thickness of the SHAM group was 570.56 ± 144.7 μm (Table S3). The endometrial thickness of the ASH group decreased in all sections and was measured as 425.28 ± 86.39 μm but it was not statistically significant (Table S3). In the ASH + HDJ group, no thickness decrease was found as much as the ASH group and the endometrial thickness was measured as 503.21 ± 50.51 μm (Table S3). Furthermore, endometrial thickness increased in the ASH + HDJ + MEL (510.65 ± 71.25 μm), ASH + HDJ + EST (454.47 ± 123.40 μm), and ASH + HDJ + MEL + EST (517.26 ± 82.79 μm) compared to the ASH group, but it was not statistically significant (Table S3).

#### 3.7.3 Cell Infiltration

The H-E staining method was applied to determine the cell infiltration, which is one of the necessary criteria, to observe the Asherman’s syndrome and the effectiveness of the substances given afterward. There was no finding suggestive of cell infiltration in the SHAM group. In all other groups, mild cell infiltration was observed but in the ASH group, more abundantly, according to the scoring. However, the differences were not statistically significant (Figure 11A–C).

#### 3.7.4 Fibrosis Level

The MT staining method was applied to determine the fibrosis. In the uterine horn sections of the SHAM group, the lamina propria, which was a loose connective tissue structure just below the surface epithelium of the endometrial layer, was observed in the normal histological structure. It was observed that the myometrium contained inner circular and outer longitudinal muscle layers, while the perimetrium contained vessels and nerves, which were the structures and features of loose connective tissue (Figure 12B, Table S4).

Findings of fibrosis were rarely observed in the uterine horn sections of the ASH group, but no statistically significant differences were found as a result of the scoring (Figure 12B, Table S4). Endometrial fibroblasts, macrophages, lymphocytes, leukocytes, and connective tissue fibers were observed in the uterine horn sections of the ASH + HDJ, ASH + HDJ + MEL, ASH + HDJ + EST, and ASH + HDJ + MEL + EST groups. No signs of fibrosis were observed in all groups, and the SHAM group was consistent with the histological structure of the endometrium connective tissue (Figure 12B, Table S4).

### 3.8 Biochemical Evaluation

In biochemical studies, six parameters, including MDA, MPO, NO, CAT, SOD, and tGSH, were investigated. MDA, MPO, and NO represent inflammation, while CAT, SOD, and tGSH represent the level of defense against inflammation

#### 3.8.1 MDA, MPO, and NO Levels

**Figure 13:**
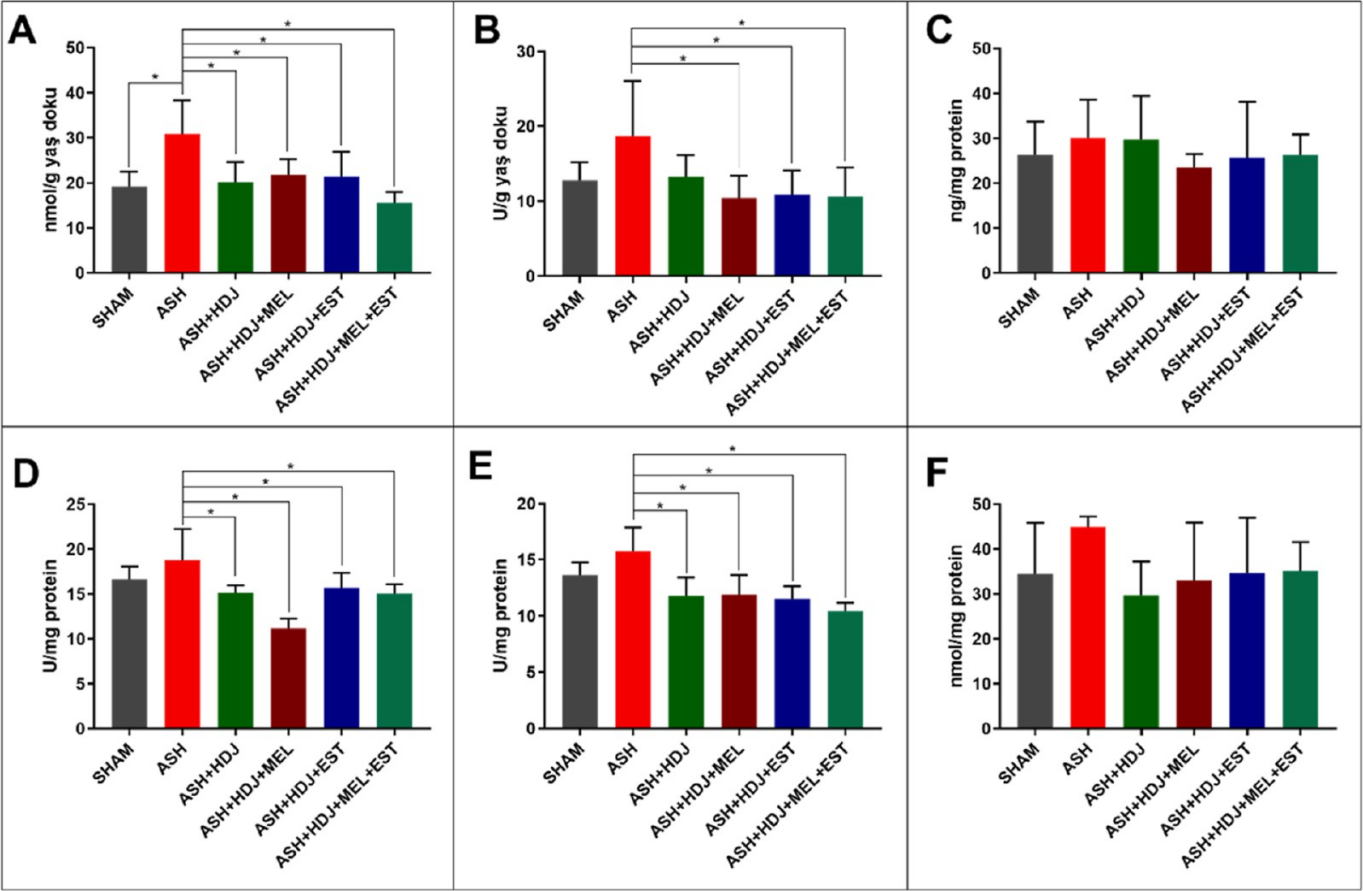
Effect of hydrogels on (A) MDA, (B) MPO, (C) NO levels and (D) CAT, (E) SOD, (F) tGSH activities in uterine horn tissues against Asherman’s damage.

MDA is an important parameter in the evaluation of tissue inflammation. As seen in Figure 13A, the MDA level of the ASH group increased significantly compared to the control (SHAM) (*p* < 0.05). It was an indication that tissue damage had occurred. On the other hand, MDA levels were significantly lower in all ASH + HDJ, ASH + HDJ + MEL, ASH + HDJ + EST, and ASH + HDJ + MEL + EST groups compared to the ASH group (*p* < 0.05). There was no difference in MDA levels between the hormone-free hydrogel and hydrogel groups containing the hormone. These results considered that the hormonal effect of the improvement is not reflected in the MDA level, and the hydrogel mechanically kept the uterine canal open. In addition, a decrease was observed in ASH + HDJ + MEL + EST group compared to ASH + HDJ + MEL and ASH + HDJ + EST, but it was not significant (*p* > 0.05).

MPO is another significant parameter in the evaluation of tissue inflammation. All hormone-containing groups except the hormone-free hydrogel group showed a significant decrease in MPO results compared to the ASH group, as illustrated in Figure 13B (*p* < 0.05). Here, the hormonal effect in healing was remarkable. However, no change was observed between hormones. The ASH group showed an increase in MPO level compared to the control group (SHAM), but this change was not significant (*p* > 0.05).

Another important parameter for determining inflammation is the measurement of NO level. In the results, the NO level of the ASH group increased compared to the SHAM group, but this increase was not significant. There was no difference between the ASH-HDJ and ASH groups while ASH + HDJ + MEL, ASH + HDJ + EST, and ASH + HDJ + MEL + EST groups showed a decrease compared to the ASH group, but these decreases were not significant (*p* > 0.05) (Figure 13C). However, it is known that melatonin protects tissues against intrauterine adhesion damage.^52^ MDA, MPO, and NO results were parallel with each other and supported the histological results.

#### 3.8.2 CAT and SOD Activity and the tGSH Level

CAT is an important enzyme in the antioxidant defense system. According to the results of CAT enzyme activity, a significant decrease was observed between the ASH group and all other groups containing hydrogel, as depicted in Figure 13D (*p* < 0.05). The low activity of the control and treatment groups against the damage group may be due to the fact that the ASH group did not cause enough damage to increase the CAT activity. On the other hand, the significant difference between the ASH group and the hydrogel groups could be interpreted as the activation of the organism’s defense mechanism since the damage in the ASH group was not very high. In addition, also no significance of the increase between the SHAM and ASH groups indicated that the level of damage was low and the defense mechanism was activated (*p* > 0.05). Here, no statistics were found between the hormone-free hydrogel and the hormone-containing groups (*p* > 0.05). This was because the absorbed hormones were released from the hydrogel rapidly (∼3 h) and could not show their effect after 14 days.

Another important enzyme in the antioxidant defense system is SOD. It functions in the organism by reducing superoxide radicals to hydrogen peroxide, a less reactive species. According to the results, although the increase in the ASH group was not significant compared to the control (*p* > 0.05), the decrease in the hydrogel-containing groups was significant compared to ASH group, as displayed in Figure 13E (*p* < 0.05). No significant difference between the hydrogel-containing groups was determined (*p* > 0.05). These results seemed parallel to CAT results and could be interpreted similarly.

Glutathione is one of the important molecules in the antioxidant defense system and generally shows similar results to CAT and SOD enzymes.^63^ As depicted in Figure 13F, an increase in glutathione level was observed in the ASH group, while a decrease was observed in the control and treatment groups. However, none of these differences were found to be significant (*p* > 0.05). According to these results, it was thought that the hydrogels mechanically kept the uterine canal open and prevented damage.

After β-CD-PEG-600-Ec hydrogel application, the immunohistological results were obtained and the statistical evaluation of these results are given in Figure S3 and Table S5. As a result, it was seen that the β-CD-PEG-600-Ec hydrogel structure was a nontoxic and injectable gel with mechanical barrier properties in preventing intrauterine adhesion.

## 4 Conclusions

In the present study, the β-CD-based multifunctional hydrogels were reported in order to prevent Asherman’s syndrome or intrauterine adhesions and improve its damage. The β-CD-based hydrogels were successfully prepared, characterized, and their potential availability on Asherman’s syndrome was investigated in rat models. The CD-based hydrogels held advantages of porous structure, high swelling, injectability, excellent biocompatibility, drug-releasing properties, and antioxidant activity. Furthermore, the β-CD-PEG-600-Ec hydrogel exhibited the most satisfactory properties rather than other ones. The potential of this hydrogel in preventing Asherman’s syndrome was evaluated in rat models. Overall, the experiment’s findings suggest that the prepared double drug-loaded β-CD-PEG-600-Ec hydrogel can potentially be used to prevent Asherman’s syndrome due to its promising properties.

## Supporting Information Available

Optimization of the reaction conditions; injectability results of the hydrogel and immunohistological staining methods and images (PDF)

## References

[ref1] Conforti A.; Alviggi C.; Mollo A.; De Placido G.; Magos A. The management of Asherman syndrome: a review of literature. Reprod. Biol. Endocrinol. 2013, 11, 118 10.1186/1477-7827-11-118. 24373209 PMC3880005

[ref2] Asherman J. G. Traumatic intra-uterine adhesions. J. Obstet. Gynaecol. Br. Emp. 1950, 57 (6), 892–896. 10.1111/j.1471-0528.1950.tb06053.x. 14804168

[ref3] Asherman J. G. Amenorrhoea traumatica (atretica). J. Obstet. Gynaecol. Br. Emp. 1948, 55 (1), 23–30. 10.1111/j.1471-0528.1948.tb07045.x. 18902559

[ref4] Netter A.; Musset R.; Lambert A.; Salomon Y.; Montbazet G. Tuberculous endo-uterine symphysis; an anatomo-clinical and radiologically characteristic syndrome. Gynecol. Obstet (Paris). 1955, 54 (1), 19–36. 14391756

[ref5] Valle R. F.; Sciarra J. J. Intrauterine adhesions: hysteroscopic diagnosis, classification, treatment, and reproductive outcome. Am. J. Obstet. Gynecol. 1988, 158 (6), 1459–1470. 10.1016/0002-9378(88)90382-1. 3381869

[ref6] Roge P.; D’Ercole C.; Cravello L.; Boubli L.; Blanc B. Hysteroscopic management of uterine synechiae: a series of 102 observations. Eur. J. Obstet. Gynecol. Reprod. Biol. 1996, 65 (2), 189–193. 10.1016/0301-2115(95)02342-9. 8730623

[ref7] Peppas N. A.; Hilt J. Z.; Khademhosseini A.; Langer R. Hydrogels in biology and medicine: from molecular principles to bionanotechnology. Adv. Mater. 2006, 18 (11), 1345–1360. 10.1002/adma.200501612.

[ref8] Hennink W. E.; van Nostrum C. F. Novel crosslinking methods to design hydrogels. Adv. Drug Delivery Rev. 2002, 54 (1), 13–36. 10.1016/S0169-409X(01)00240-X. 11755704

[ref9] Hoffman A. S. Hydrogels for biomedical applications. Adv. Drug Delivery Rev. 2012, 64, 18–23. 10.1016/j.addr.2012.09.010. 11755703

[ref10] Barbucci R. Hydrogels: Biological Properties and Applications; Springer-Verlag Mailand: Italia, 2009.

[ref11] Caló E.; Khutoryanskiy V. V. Biomedical applications of hydrogels: A review of patents and commercial products. Eur. Polym. J. 2015, 65, 252–267. 10.1016/j.eurpolymj.2014.11.024.

[ref12] Brandl F.; Sommer F.; Goepferich A. Rational design of hydrogels for tissue engineering: impact of physical factors on cell behavior. Biomaterials 2007, 28 (2), 134–146. 10.1016/j.biomaterials.2006.09.017. 17011028

[ref13] Fedorovich N. E.; Alblas J.; de Wijn J. R.; Hennink W. E.; Verbout A. J.; Dhert W. J. Hydrogels as extracellular matrices for skeletal tissue engineering: state-of-the-art and novel application in organ printing. Tissue Eng. 2007, 13 (8), 1905–1925. 10.1089/ten.2006.0175. 17518748

[ref14] Hoare T. R.; Kohane D. S. Hydrogels in drug delivery: Progress and challenges. Polymer 2008, 49 (8), 1993–2007. 10.1016/j.polymer.2008.01.027.

[ref15] Abbott J.; Thomson A.; Vancaillie T. SprayGel following surgery for Asherman’s syndrome may improve pregnancy outcome. J. Obstet. Gynaecol. 2004, 24 (6), 710–711. 10.1080/01443610400008206. 16147625

[ref16] Muller S. A.; Weis C.; Odermatt E. K.; Knaebel H. P.; Wente M. N. A hydrogel for adhesion prevention: characterization and efficacy study in a rabbit uterus model. Eur. J. Obstet. Gynecol. Reprod. Biol. 2011, 158 (1), 67–71. 10.1016/j.ejogrb.2010.11.008. 21146281

[ref17] Nijenhuis R. J.; Smeets A. J.; Morpurgo M.; Boekkooi P. F.; Reuwer P. J.; Smink M.; van Rooij W. J.; Lohle P. N. Uterine artery embolisation for symptomatic adenomyosis with polyzene F-coated hydrogel microspheres: three-year clinical follow-up using UFS-QoL questionnaire. Cardiovasc. Intervent. Radiol. 2015, 38 (1), 65–71. 10.1007/s00270-014-0878-1. 24692030

[ref18] Gao X.; Deng X.; Wei X.; Shi H.; Wang F.; Ye T.; Shao B.; Nie W.; Li Y.; Luo M.; Gong C.; Huang N. Novel thermosensitive hydrogel for preventing formation of abdominal adhesions. Int. J. Nanomed. 2013, 8, 2453–2463. 10.2147/IJN.S46357. PMC3716558 23885172

[ref19] Navath R. S.; Menjoge A. R.; Dai H.; Romero R.; Kannan S.; Kannan R. M. Injectable PAMAM dendrimer-PEG hydrogels for the treatment of genital infections: formulation and in vitro and in vivo evaluation. Mol. Pharmaceutics 2011, 8 (4), 1209–1223. 10.1021/mp200027z. PMC3556449 21615144

[ref20] Khade S. M.; Behera B.; Sagiri S. S.; Singh V. K.; Thirugnanam A.; Pal K.; Ray S. S.; Pradhan D. K.; Bhattacharya M. K. Gelatin-PEG based metronidazole-loaded vaginal delivery systems: preparation, characterization and in vitro antimicrobial efficiency. Iran Polym. J. 2014, 23 (3), 171–184. 10.1007/s13726-013-0213-8.

[ref21] Frank L. A.; Sandri G.; D’Autilia F.; Contri R. V.; Bonferoni M. C.; Caramella C.; Frank A. G.; Pohlmann A. R.; Guterres S. S. Chitosan gel containing polymeric nanocapsules: a new formulation for vaginal drug delivery. Int. J. Nanomed. 2014, 9, 3151–3161. 10.2147/ijn.s62599. PMC4085301 25061292

[ref22] Li N.; Yu M.; Deng L.; Yang J.; Dong A. Thermosensitive hydrogel of hydrophobically-modified methylcellulose for intravaginal drug delivery. J. Mater. Sci. Mater. Med. 2012, 23 (8), 1913–1919. 10.1007/s10856-012-4664-9. 22569735

[ref23] Li W. Z.; Zhao N.; Zhou Y. Q.; Yang L. B.; Xiao-Ning W.; Bao-Hua H.; Peng K.; Chun-Feng Z. Post-expansile hydrogel foam aerosol of PG-liposomes: a novel delivery system for vaginal drug delivery applications. Eur. J. Pharm. Sci. 2012, 47 (1), 162–169. 10.1016/j.ejps.2012.06.001. 22705561

[ref24] Liu D.; Yun Y.; Yang D.; Hu X.; Dong X.; Zhang N.; Zhang L.; Yin H.; Duan W. What Is the Biological Function of Uric Acid? An Antioxidant for Neural Protection or a Biomarker for Cell Death. Dis. Markers. 2019, 2019, 1–9. 10.1155/2019/4081962. PMC6348815 30733836

[ref25] Zhang S.-S.; Xu X.-X.; Xiang W.-W.; Zhang H.-H.; Lin H.-L.; Shen L.-E.; Lin Q.; Lin F.; Zhou Z.-Y. Using 17β-estradiol heparin-poloxamer thermosensitive hydrogel to enhance the endometrial regeneration and functional recovery of intrauterine adhesions in a rat model. FASEB J. 2020, 34 (1), 446–457. 10.1096/fj.201901603RR. 31914682

[ref26] Wenbo Q.; Lijian X.; Shuangdan Z.; Jiahua Z.; Yanpeng T.; Xuejun Q.; Xianghua H.; Jingkun Z. Controlled releasing of SDF-1α in chitosan-heparin hydrogel for endometrium injury healing in rat model. Int. J. Biol. Macromol. 2020, 143, 163–172. 10.1016/j.ijbiomac.2019.11.184. 31765745

[ref27] Yao Q.; Zheng Y. W.; Lan Q. H.; Wang L. F.; Huang Z. W.; Chen R.; Yang Y.; Xu H. L.; Kou L.; Zhao Y. Z. Aloe/poloxamer hydrogel as an injectable β-estradiol delivery scaffold with multi-therapeutic effects to promote endometrial regeneration for intrauterine adhesion treatment. Eur. J. Pharm. Sci. 2020, 148, 105316 10.1016/j.ejps.2020.105316. 32201342

[ref28] Xu H. L.; Xu J.; Zhang S. S.; Zhu Q. Y.; Jin B. H.; ZhuGe D. L.; Shen B. X.; Wu X. Q.; Xiao J.; Zhao Y. Z. Temperature-sensitive heparin-modified poloxamer hydrogel with affinity to KGF facilitate the morphologic and functional recovery of the injured rat uterus. Drug Delivery 2017, 24 (1), 867–881. 10.1080/10717544.2017.1333173. 28574291 PMC8241134

[ref29] Jiang P.; Tang X.; Wang H.; Dai C.; Su J.; Zhu H.; Song M.; Liu J.; Nan Z.; Ru T.; Li Y.; Wang J.; Yang J.; Chen B.; Dai J.; Hu Y. Collagen-binding basic fibroblast growth factor improves functional remodeling of scarred endometrium in uterine infertile women: A pilot study. Sci. China Life Sci. 2019, 62 (12), 1617–1629. 10.1007/s11427-018-9520-2. 31515729

[ref30] Liu Y.; Cai J.; Luo X.; Wen H.; Luo Y. Collagen scaffold with human umbilical cord mesenchymal stem cells remarkably improves intrauterine adhesions in a rat model. Gynecol. Obstet. Invest. 2020, 85 (3), 267–276. 10.1159/000505691. 32289792

[ref31] Liu Y. R.; Liu B.; Yang B. P.; Lan Y.; Chi Y. G. Efficacy of hyaluronic acid on the prevention of intrauterine adhesion and the improvement of fertility: A meta-analysis of randomized trials. Complement. Ther. Clin. Pract. 2022, 47, 101575 10.1016/j.ctcp.2022.101575. 35349823

[ref32] Zhou Q.; Shi X.; Saravelos S.; Huang X.; Zhao Y.; Huang R.; Xia E.; Li T. C. AutoCross-linked hyaluronic acid gel for prevention of intrauterine adhesions after hysteroscopic adhesiolysis: A randomized controlled trial. J. Minim. Invasive Gynecol. 2021, 28 (2), 307–313. 10.1016/j.jmig.2020.06.030. 32681996

[ref33] Wu F.; Lei N.; Yang S.; Zhou J.; Chen M.; Chen C.; Qiu L.; Guo R.; Li Y.; Chang L. Treatment strategies for intrauterine adhesion: focus on the exosomes and hydrogels. Front. Bioeng. Biotechnol. 2023, 11, 1264006 10.3389/fbioe.2023.1264006. 37720318 PMC10501405

[ref34] Liu Z.; Wang L.; Bao C.; Li X.; Cao L.; Dai K.; Zhu L. Cross-Linked PEG via Degradable Phosphate Ester Bond: Synthesis, Water-Swelling, and Application as Drug Carrier. Biomacromolecules 2011, 12 (6), 2389–2395. 10.1021/bm2004737. 21563838

[ref35] Zhang L.; Jeong Y. I.; Zheng S.; Kang D. H.; Suh H.; Kim I. Crosslinked poly(ethylene glycol) hydrogels with degradable phosphamide linkers used as a drug carrier in cancer therapy. Macromol. Biosci. 2014, 14 (3), 401–410. 10.1002/mabi.201300327. 24821668

[ref36] Teng X.; Xu H.; Song W.; Shi J.; Xin J.; Hiscox W. C.; Zhang J. Preparation and Properties of Hydrogels Based on PEGylated Lignosulfonate Amine. ACS Omega 2017, 2 (1), 251–259. 10.1021/acsomega.6b00296. 31457225 PMC6641139

[ref37] Khodaverdi E.; Heidari Z.; Tabassi S. A.; Tafaghodi M.; Alibolandi M.; Tekie F. S.; Khameneh B.; Hadizadeh F. Injectable supramolecular hydrogel from insulin-loaded triblock PCL-PEG-PCL copolymer and gamma-cyclodextrin with sustained-release property. AAPS PharmSciTechnol. 2015, 16 (1), 140–149. 10.1208/s12249-014-0198-4. PMC4309819 25224297

[ref38] Liu M.; Kono K.; Fréchet J. M. J. Water-soluble dendritic unimolecular micelles:. J. Controlled Release 2000, 65 (1–2), 121–131. 10.1016/S0168-3659(99)00245-X. 10699276

[ref39] Salmaso S.; Semenzato A.; Bersani S.; Matricardi P.; Rossi F.; Caliceti P. Cyclodextrin/PEG based hydrogels for multi-drug delivery. Int. J. Pharm. 2007, 345 (1–2), 42–50. 10.1016/j.ijpharm.2007.05.035. 17597313

[ref40] Yavaşer R.; Girgin B.; Korkmaz O.; Karagözler A. A. Production and drug release assesment of melatonin-loaded alginate/gum arabic beads. J. Turk. Chem. Soc., Sect. A: Chem. 2016, 3 (3), 205–216. 10.18596/jotcsa.30880.

[ref41] Jung B. O.; Chung S. J.; Lee S. B. Preparation and characterization of eugenol-grafted chitosan hydrogels and their antioxidant activities. J. Appl. Polym. Sci. 2006, 99 (6), 3500–3506. 10.1002/app.22974.

[ref42] Hillegass L. M.; Griswold D. E.; Brickson B.; Albrightson-Winslow C. Assessment of myeloperoxidase activity in whole rat kidney. J. Pharmacol. Methods. 1990, 24 (4), 285–295. 10.1016/0160-5402(90)90013-B. 1963456

[ref43] Luck H. Catalase. Methods of Enzymatic Analysis, 2nd ed.; Academic Press: New York, 1963.

[ref44] McCord J. M.; Fridovich I. Superoxide Dismutase. J. Biol. Chem. 1969, 244 (22), 6049–6055. 10.1016/S0021-9258(18)63504-5. 5389100

[ref45] Akerboom T. P.; Sies H. [48] Assay of glutathione, glutathione disulfide, and glutathione mixed disulfides in biological samples. Methods Enzymol. 1981, 77, 373–382. 10.1016/S0076-6879(81)77050-2. 7329314

[ref46] Buege J. A.; Aust S. D. [30] Microsomal lipid peroxidation. Methods Enzymol. 1978, 52, 302–310. 10.1016/S0076-6879(78)52032-6. 672633

[ref47] Zhou Q.; Zhong L.; Wei X.; Dou W.; Chou G.; Wang Z. Baicalein and hydroxypropyl-gamma-cyclodextrin complex in poloxamer thermal sensitive hydrogel for vaginal administration. Int. J. Pharm. 2013, 454 (1), 125–134. 10.1016/j.ijpharm.2013.07.006. 23850236

[ref48] Lin X.; Wei M.; Li T. C.; Huang Q.; Huang D.; Zhou F.; Zhang S. A comparison of intrauterine balloon, intrauterine contraceptive device and hyaluronic acid gel in the prevention of adhesion reformation following hysteroscopic surgery for Asherman syndrome: a cohort study. Eur. J. Obstet. Gynecol. Reprod. Biol. 2013, 170 (2), 512–516. 10.1016/j.ejogrb.2013.07.018. 23932377

[ref49] Can S.; Kirpinar G.; Dural O.; Karamustafaoglu B. B.; Tas I. S.; Yasa C.; Ugurlucan F. G. Efficacy of a New Crosslinked Hyaluronan Gel in the Prevention of Intrauterine Adhesions. JSLS. 2018, 22 (4), e2018.00036 10.4293/jsls.2018.00036. PMC6261745 30524185

[ref50] Li X.; Wu L.; Zhou Y.; Fan X.; Huang J.; Wu J.; Yu R.; Lou J.; Yang M.; Yao Z.; Xue M. New Crosslinked Hyaluronan Gel for the Prevention of Intrauterine Adhesions after Dilation and Curettage in Patients with Delayed Miscarriage: A Prospective, Multicenter, Randomized, Controlled Trial. J. Minim. Invasive. Gynecol. 2019, 26 (1), 94–99. 10.1016/j.jmig.2018.03.032. 29678756

[ref51] Pabuccu E. G.; Kovanci E.; Sahin O.; Arslanoglu E.; Yildiz Y.; Pabuccu R. New Crosslinked Hyaluronan Gel, Intrauterine Device, or Both for the Prevention of Intrauterine Adhesions. JSLS. 2019, 23 (1), e2018.00108 10.4293/jsls.2018.00108. PMC6400248 30846896

[ref52] Lee D. Y.; Lee S. R.; Kim S. K.; Joo J. K.; Lee W. S.; Shin J. H.; Cho S.; Park J. C.; Kim S. H. A New Thermo-Responsive Hyaluronic Acid Sol-Gel to Prevent Intrauterine Adhesions after Hysteroscopic Surgery: A Randomized, Non-Inferiority Trial. Yonsei Med. J. 2020, 61 (10), 868–874. 10.3349/ymj.2020.61.10.868. 32975061 PMC7515784

[ref53] Tafti S. Z. G.; Javaheri A.; Firoozabadi R. D.; Ashkezar S. K.; Abarghouei H. F. Role of hyaluronic acid intrauterine injection in the prevention of Asherman’s syndrome in women undergoing uterine septum resection: An RCT. Int. J. Reprod. Biomed. 2021, 19 (4), 339–346. 10.18502/ijrm.v19i4.9060. 33997593 PMC8106814

[ref54] Feng M.; Hu S.; Qin W.; Tang Y.; Guo R.; Han L. Bioprinting of a Blue Light-Cross-Linked Biodegradable Hydrogel Encapsulating Amniotic Mesenchymal Stem Cells for Intrauterine Adhesion Prevention. ACS Omega 2021, 6 (36), 23067–23075. 10.1021/acsomega.1c02117. 34549107 PMC8444209

[ref55] Fuchs N.; Smorgick N.; Ben Ami I.; Vaknin Z.; Tovbin Y.; Halperin R.; Pansky M. Intercoat (Oxiplex/AP gel) for preventing intrauterine adhesions after operative hysteroscopy for suspected retained products of conception: double-blind, prospective, randomized pilot study. J. Minim. Invasive. Gynecol. 2014, 21 (1), 126–130. 10.1016/j.jmig.2013.07.019. 23954387

[ref56] Reiter R. J.; Tan D.-x.; Osuna C.; Gitto E. Actions of melatonin in the reduction of oxidative stress. J. Biomed. Sci. 2000, 7 (6), 444–458. 10.1007/BF02253360. 11060493

[ref57] Wang J.; Yang C.; Xie Y.; Chen X.; Jiang T.; Tian J.; Hu S.; Lu Y. Application of Bioactive Hydrogels for Functional Treatment of Intrauterine Adhesion. Front. Bioeng. Biotechnol. 2021, 9, 760943 10.3389/fbioe.2021.760943. 34621732 PMC8490821

[ref58] Chen Y.; Fei W.; Zhao Y.; Wang F.; Zheng X.; Luan X.; Zheng C. Sustained delivery of 17β-estradiol by human amniotic extracellular matrix (HAECM) scaffold integrated with PLGA microspheres for endometrium regeneration. Drug Delivery 2020, 27, 1165–1175. 10.1080/10717544.2020.1801891. 32755258 PMC7470125

[ref59] Saat N.; Risvanli A.; Dogan H.; Onalan E.; Akpolat N.; Seker I.; Sahna E. Effect of melatonin on torsion and reperfusion induced pathogenesis of rat uterus. Biotechnol. Histochem. 2019, 94, 533–539. 10.1080/10520295.2019.1605456. 31070494

[ref60] Lee J. Y.; Song H.; Dash O.; Park M.; Shin N. E.; McLane M. W.; Lei J.; Hwang J. Y.; Burd I. Administration of melatonin for prevention of preterm birth and fetal brain injury associated with premature birth in a mouse model. Am. J. Reprod. Immunol. 2019, 82, e13151 10.1111/aji.13151. 31131935

[ref61] Torabi H.; Mehdikhani M.; Varshosaz J.; Shafiee F. An innovative approach to fabricate a thermosensitive melatonin-loaded conductive pluronic/chitosan hydrogel for myocardial tissue engineering. J. Appl. Polym. Sci. 2021, 138, 50327 10.1002/app.50327.

[ref62] Xiao L.; Lin J.; Chen R.; Huang Y.; Liu Y.; Bai J.; Ge G.; Shi X.; Chen Y.; Shi J.; Aiqing L.; Yang H.; Geng D.; Wang Z. Sustained Release of Melatonin from GelMA Liposomes Reduced Osteoblast Apoptosis and Improved Implant Osseointegration in Osteoporosis. Oxid. Med. Cell. Longev. 2020, 2020, 1–20. 10.1155/2020/6797154. PMC7275204 32566094

[ref63] Ighodaro O. M.; Akinloye O. A. First line defence antioxidants-superoxide dismutase (SOD), catalase (CAT) and glutathione peroxidase (GPX): Their fundamental role in the entire antioxidant defence grid. Alexandria J. Med. 2018, 54, 287–293. 10.1016/j.ajme.2017.09.001.

