# Supplementary material for: Synthesis of Cyclodextrin-Based Multifunctional Biocompatible Hydrogels and Their Use in the Prevention of Intrauterine Adhesions (Asherman’s Syndrome) after Surgical Injury
Source: ACS Omega. 2024 Jul 11;9(29):31957–73. doi: 10.1021/acsomega.4c03655 (PMC11270706; doi:10.1021/acsomega.4c03655)
Supplement: Supplementary file 1 — ao4c03655_si_001.pdf [file ao4c03655_si_001.pdf]

Synthesis of cyclodextrin-based multi-functional biocompatible hydrogels and their use in the prevention of intrauterine adhesions (Asherman's syndrome) after surgical injury

Busra Aksoy Erden[1], Meltem Kurus[2], Ilgin Turkcuoglu[3], Rauf Melekoglu[4], Sevgi Balcioglu[5,6], Birgul Yigitcan[7], Burhan Ates[6*], Suleyman Koytepe[6*]

[1] Bartın University, Central Research Laboratory Application and Research Center, Bartın, Turkey

[2] İzmir Katip Çelebi University, Faculty of Medicine, Department of Histology and Embryology, İzmir, Turkey

[3] SANKO University, Faculty of Medicine, Department of Obstetrics and Gynecology, Gaziantep, Turkey

[4] İnönü University, Faculty of Medicine, Department of Obstetrics and Gynecology, Malatya, Turkey

[5] Sakarya University of Applied Sciences, Department of Medicinal Laboratory, Sakarya, Turkey

[6] İnönü University, Faculty of Science and Literature, Department of Chemistry, Malatya, Turkey

[7] İnönü University, Faculty of Medicine, Department of Histology and Embryology, Malatya, Turkey

***Corresponding Author 1:** Prof. Burhan ATES

Address: İnönü University, Faculty of Science and Literature, Department of Chemistry, Malatya, Turkey

E-mail: burhan.ates@inonu.edu.tr

ORCID ID: 0000-0001-6080-229X

***Corresponding Author 2:** Prof. Suleyman KOYTEPE

Address: İnönü University, Faculty of Science and Literature, Department of Chemistry, Malatya, Turkey

E-mail: suleyman.koytepe@inonu.edu.tr

ORCID ID: 0000-0002-4788-278X

Determination of Monomer Ratios for Hydrogel Synthesis and Optimization of Hydrogel Synthesis Temperature

To optimize the monomer ratios to be used before proceeding with the synthesis studies within the scope of the project, polyphenol, polyethylene glycol diglycidyl ether and cyclodextrin groups as cross-linking groups were first studied in different ratios. In this study, optimum monomer ratios were determined. Within the scope of the project, as a preliminary trial, polyethylene glycol diglycidyl ether, cyclodextrin, and polyphenol molar ratios; 96:2:2, 90:3:5, 92:4:4and 90/4/6, differently for β-cyclodextrins. Some of these trials are given in Figure S1. According to the FTIR result, the ratio 90/4/6 was chosen as the optimum ratio.

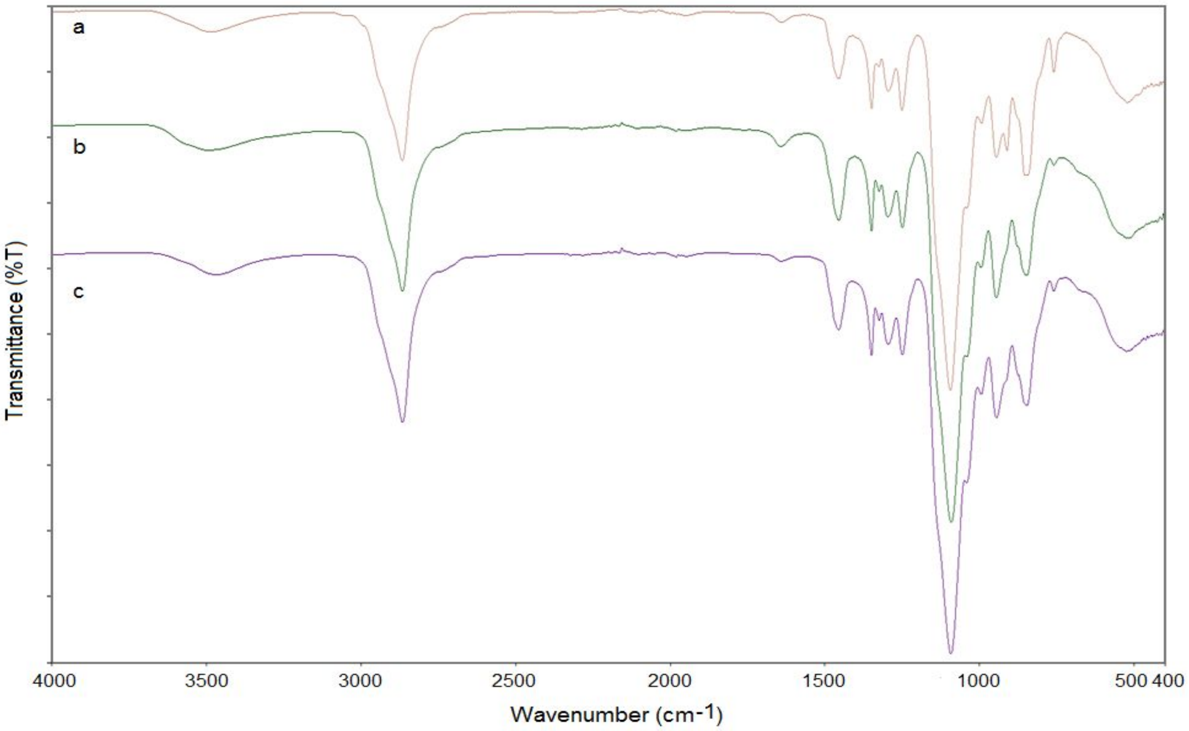

Figure S1. FTIR spectra of hydrogel structures obtained at different monomer ratios (polyethylene glycol diglycidyl ether:cyclodextrin:polyphenol ratios; a; 96:2:2, b; 90:3:5, c; 92:4:4)

As a second optimization parameter, the effect of temperature on hydrogel synthesis was studied. Synthesis temperature in hydrogel synthesis; It was carried out at 80°C, 70°C, 60°C, 50°C, 40°C, 30°C and 20°C, and gelation time, gel formation and gel composition analyzes were performed. In line with these analyses, the desired hydrogel structure was achieved within the optimum temperature. IR spectra of gel experiments obtained during these preliminary experiments are given in figure S2. Another important parameter in hydrogel synthesis is the reaction temperature. For this reason, the reaction temperature was optimized

before proceeding with serial synthesis within the scope of the study. FTIR spectra of repeated syntheses at 20, 30, 40, 50, 60, 70 and 80 °C and temperatures are shown in Figure S2. Within the scope of the study, hydrogel synthesis was attempted at as low temperatures as possible. During these experiments, 60°C was preferred as the optimum synthesis temperature. In negative experiments, solid or near-solid products were obtained or no binding occurred. The main goal in optimization is the formation of semi-flexible hydrogel structured products. After the reaction, the lowest temperature and monomer ratio at which this type of gel-structured product was obtained were chosen and characterized as optimum.

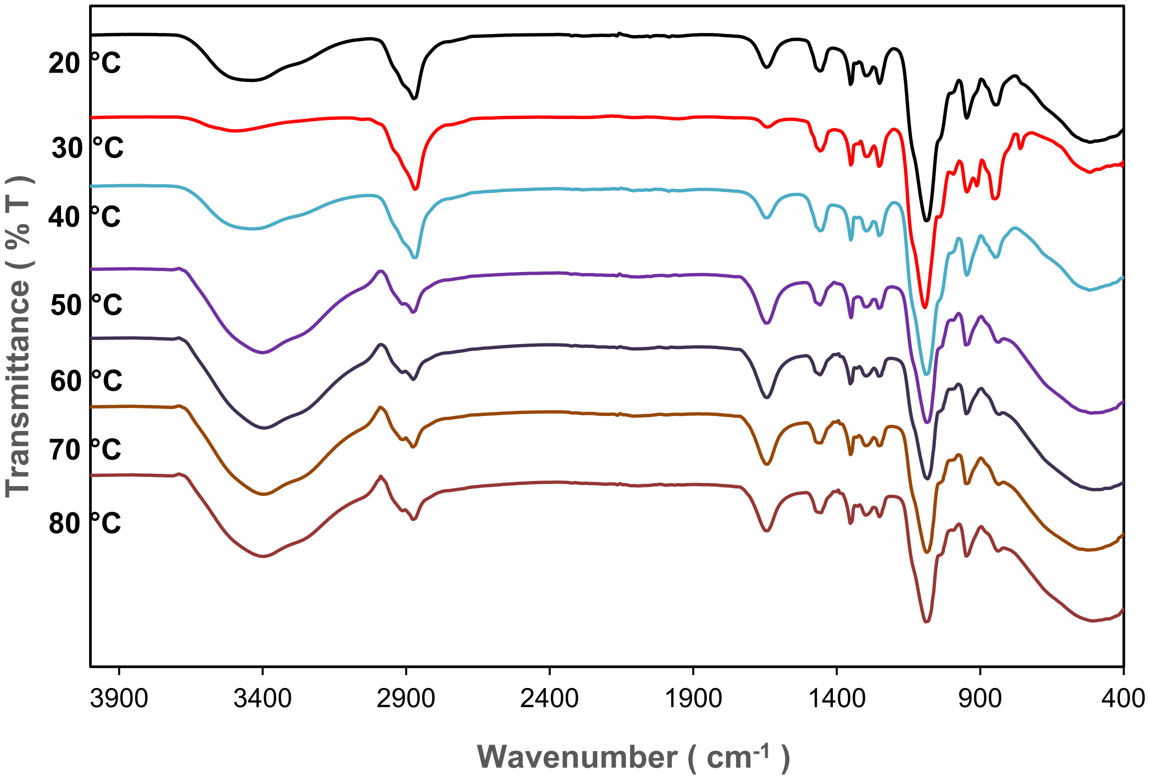

Figure S2. FTIR spectra of hydrogel structures obtained at different temperatures

Table S1. Injectability test results of β-CD-PEG-600 hydrogels from different polyphenols.

	18G	20G	22G	24G	26G
β-CD-PEG-600-CA	-	-	-	-	-
β-CD-PEG-600-GA	+	+	+	+	-
β-CD-PEG-600-Ec	+	+	+	+	+
β-CD-PEG-600-Quer	+	+	+	+	+
β-CD-PEG-600-Cur	+	+	+	+	+

Table S2. Comparison of damage parameters of uterine horn tissues

Groups	Epithelial damage median (min.-max.)	Epithelial desquamation median (min.-max.)	Congestion median (min.-max.)
SHAM	0 (0-1)[a]	0 (0-2)	0 (0-2)[a]
ASH	3 (2-3)[b]	1 (0-2)	2 (1-3)[b]
ASH+HDJ	2 (1-3)[c,e]	0 (0-2)	1 (0-2)[c,d]
ASH+HDJ+MEL	1 (0-2)[d,e]	0 (0-2)	1 (0-2)[c,d]
ASH+HDJ+EST	2 (1-3)[b,c]	0 (0-2)	1 (0-2)[b,c]
ASH+HDJ+MEL+EST	0 (0-2)[a,d]	0 (0-1)	0 (0-2)[a,d]
p-value	<0.001	0.531	0.003

Table S3. Comparison of endometrial thickness between groups

Groups	N	Mean	Std. Deviation	P
SHAM	7	570.56	144.47	0.122
ASH	7	425.28	86.39	
ASH+HDJ	7	503.21	50.51	
ASH+HDJ+MEL	7	510.65	71.25	
ASH+HDJ+EST	7	454.47	123.40	
ASH+HDJ+MEL+EST	7	517.26	82.79	

Table S4. Fibrosis and inflammation

Groups	Fibrosis	Inflammation
SHAM	0 (0-1)	0 (0-0)
ASH	0 (0-1)	0 (0-2)
ASH+HDJ	0 (0-1)	0 (0-1)
ASH+HDJ+MEL	0 (0-1)	0 (0-1)
ASH+HDJ+EST	0 (0-0)	0 (0-0)
ASH+HDJ+MEL+EST	0 (0-0)	0 (0-0)

Immunohistochemical evaluation

The statistical evaluation of the immunohistochemical staining results of the uterine sections of all groups is presented in Table S5. According to the thickness of the myometrium, only the ASH group was seen as different from all other groups. No significant difference was observed between the other groups.

Table S5. Immunohistochemical staining test results (median (min.max))

Group	Myometrium Thickness	HOXA10	KI-67	VEGF
SHAM	121.17 (103.98-167.47) a	1 (0-2) a	1 (0-2) a,c	1 (0-2) a
ASH	108.79 (102.6-131.24) b	1 (0-2) a	2.5 (1-3) b	2 (2-3) b
ASH+HDJ	130.57 (120.64-173.72) a	1 (0-2) a	2 (1-3) b	2.5 (2-3) b
ASH+HDJ+MEL	139.88 (120.69-209.5) a	2 (1-3) b	2 (0-2) a,b,c	2 (1-3) b
ASH+HDJ+EST	142.19 (97.39-157.8) a	2 (1-3) b	2 (1-3) b	2 (2-3) b
ASH+HDJ+MEL+EST	152.38 (109.29-177.92) a	1 (0-2) a,b	1 (0-3) c	1 (0-2) a
p- value	0.015	0.009	0.015	<0.001

* The difference between groups with different superscripts was statistically significant.

According to VEGF staining, SHAM-ASH, SHAM-ASH+HDJ, SHAM-ASH+HDJ+MEL, SHAM-ASH+HDJ+EST, ASH-ASH+HDJ+MEL, ASH+HDJ-ASH+HDJ+MEL, ASH+HDJ+MEL-ASH+HDJ+MEL, and ASH+HDJ+EST-ASH+HDJ+MEL groups were determined statistically different. In HoxA10 staining, SHAM-ASH+HDJ+EST, ASH-ASH+HDJ+EST, SHAM-ASH+HDJ+MEL, SHAM-ASH+HDJ+MEL, ASH+HDJ-ASH+HDJ+MEL, and ASH+HDJ-ASH+HDJ+EST groups were statistically different. In Ki67 staining, SHAM-ASH, SHAM-ASH+HDJ, SHAM-ASH+HDJ+EST, ASH-ASH+HDJ+MEL+EST, ASH+HDJ-ASH+HDJ+MEL, and ASH+HDJ+EST-ASH+HDJ+MEL groups were detected statistically different.

a. VEGF immunohistochemical staining

In the uterus sections belonging to SHAM group, the staining was intense in the endometrium and glandular epithelial cells, while the staining in the vascular endothelium and stroma was moderate (Figure S3A). In ASH group, intense staining was observed in the apical regions of the endometrial epithelium and glandular epithelial cells, cells in the stroma, and vascular endothelium. While the intensity of VEGF staining of the endometrium and glandular epithelium in the uterus sections belonging to ASH+HDJ group was almost the same as that of

the ASH group, it was observed that the staining was less in the stromal cells. In addition, significant staining was observed in the vascular endothelium. Intense staining was observed in the uterine sections belonging to ASH+HDJ+MEL group, in the apical parts of the endometrium and gland epithelium. The staining in the stroma was very weak. Moderate staining was detected in the vascular endothelium. Intense staining, especially in vascular endothelium, was quite evident in the uterine sections of ASH+HDJ+EST group. While the staining in the endometrial epithelium and lamina propria areas under the epithelium was close to the ASH group, the staining gradually decreased in the deeper and myometrial layers. Staining intensity continued in the gland epithelium. In the uterine sections belonging to ASH+HDJ+MEL+EST group, the staining intensity of the endometrium, gland, and vascular epithelium was similar to that of the SHAM group. A significant decrease was observed in the stromal staining intensity.

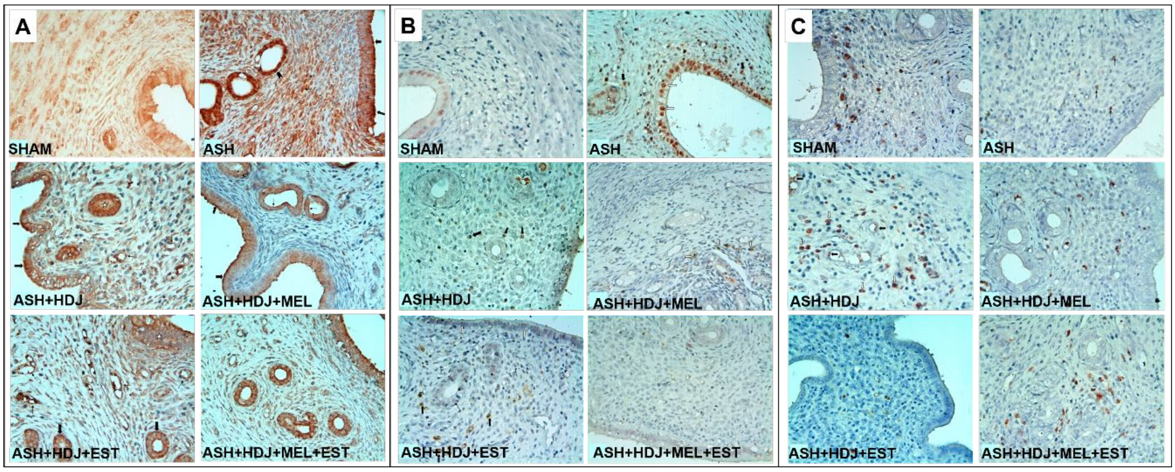

Figure S3. A) SHAM; VEGF x40, ASH; VEGFx40, thin arrow: stromal cells, thick arrow: endometrial epithelium, arrowhead: glandular epithelial cell, ASH+HDJ;VEGFx40; thick arrow: endo. epithelium, thin arrow: endothelium, star: glandular epithelium, ASH+HDJ+MEL; VEGFx40; thick arrow: endo. epithelium, thin arrow: glandular epithelium, ASH+HDJ+EST; VEGFx40; thick arrow; glandular epithelium, thin arrow: vascular endothelium, star: L. propria and stromal area, ASH+HDJ+MEL+EST; VEGFx40, B) SHAM; Ki-67x40, ASH; Ki-67x40. Thick white arrow: nuclear staining in the epithelium, thin arrow: endothelium, star: glandular epithelium, thick black arrow: stromal cells), ASH+HDJ; Ki-67x 40 thick arrow: stromal cell, thin arrow: epithelial cells, ASH+HDJ+MEL; Ki-67x40, arrow: stromal stained cells, ASH+HDJ+EST; Ki67x40. Thick black arrow: stromal cells, thick white arrow: luminal epithelial nucleus, thin arrow: glandular epithelium, ASH+HDJ+MEL+EST; Ki-67x40 and C) SHAM; HOXA-10x409, ASH; HOXA-10x40 thin arrow: stromal cells, ASH+HDJ; HOXA-10x40, white arrow: stromal cells, black arrow: edothelial cells, ASH+HDJ+MEL; HOXA-10x40, ASH+HDJ+EST; HOXA-10x40 and ASH+HDJ+MEL+EST;HOXA-10x40

b. KI-67 immunohistochemical staining

In the uterine sections belonging to the SHAM group, mild staining was observed only in the endometrial epithelium, and the staining was observed at the nuclear base (Figure S3B). In the ASH group, moderate staining was observed in the stromal cells of the luminal epithelium and the underlying lamina propria. In addition, moderate staining was observed in gland epithelium and endothelial cells of small-diameter vessels in the stroma. In ASH+HDJ group, the intensity of the endometrial epithelial staining with Ki-67 was quite weak, while the staining was more pronounced in only some stromal cells. No staining was observed in the vascular endothelium and glandular epithelium. While no cellular staining was observed belonging to ASH+HDJ+MEL group, endometrium, gland, and vascular epithelium, only some cells in the stroma were stained. Intense staining was observed in ASH+HDJ+EST group, especially in the endometrial epithelial nuclei. However, the staining in the gland epithelium was not as intense as the luminal epithelium, but moderate-intensity was observed. In stromal cells, on the other hand, there was light-intensity staining similar to that of the ASH+HDJ group. In ASH+HDJ+MEL+EST group, the staining intensity of the endometrium and glandular epithelium was extremely light. Staining was almost absent in the stromal and vascular endothelium.

c. HOXa-10 immunohistochemical staining

In the uterine sections belonging to SHAM group, while the staining of the endometrium and glandular epithelial cells was very mild, moderate staining was observed in the vascular endothelium. In stromal cells, the staining was moderate-intense and tile (brown) in color (Figure S3C). In the ASH group, only a small number of cell staining occurred in the endometrial stroma. In the ASH+HDJ group, the endometrium and glandular epithelium stained mildly with HOXA-10, while significant staining was observed in the stromal cells and vascular endothelium. In the ASH+HDJ+MEL group, no staining was observed in the endometrial epithelium, while staining was particularly evident in the vascular endothelium and stromal cells. There was also mild staining in the cytoplasm of the gland epithelial cells. Staining was observed only in the stromal cells of the uterus sections belonging to ASH+HDJ+EST group. Staining intensity was slightly better than the ASH group. In ASH+HDJ+MEL+EST group, mild staining was observed in the endometrium and glandular epithelium, while staining in the stromal cells and the entire endometrium was similar to the SHAM group.
